# PROCEED v6.1: Phenotypic rates of change evolutionary and ecological database

**DOI:** 10.1002/ecy.70009

**Published:** 2025-03-03

**Authors:** Lucas D. Gorné, Andrew P. Hendry, Fanie Pelletier, Sarah Sanderson, Cristian Correa, Carlos Arias, Marc‐Olivier Beausoleil, Maryse Boisjoly, Erika Crispo, Daniel Berner, Luis F. De León, Joseph D. DiBattista, Grant E. Haines, Benjamin C. Haller, Michael T. Kinnison, Shahin Muttalib, Ann E. McKellar, Rose E. O'Dea, Winer Daniel Reyes‐Corral, Yanny Ritchot, Krista B. Oke, Zachary T. Wood, Thomas Farrugia, Kiyoko M. Gotanda

**Affiliations:** ^1^ Department of Biological Sciences Brock University St. Catharines Ontario Canada; ^2^ Department of Biology and Redpath Museum McGill University Montréal Québec Canada; ^3^ Département de Biologie Université de Sherbrooke Sherbrooke Quebec Canada; ^4^ Instituto de Conservación Biodiversidad y Territorio, Facultad de Ciencias Forestales y Recursos Naturales Universidad Austral de Chile Valdivia Chile; ^5^ Centro de Humedales Río Cruces Universidad Austral de Chile Valdivia Chile; ^6^ School of Biology and Ecology Maine Center for Genetics in the Environment University of Maine Orono Maine USA; ^7^ Department of Zoology University of Cambridge Cambridge UK; ^8^ Present address: Facultad de Ciencias Exactas Físicas y Naturales Universidad Nacional de Córdoba Córdoba Argentina; ^9^ Present address: Consejo Nacional de Investigaciones Científicas y Técnicas, CONICET, IMBiV Córdoba Argentina; ^10^ Present address: Data Science Lab Smithsonian Tropical Research Institute (STRI) Panama Panama; ^11^ Present address: Department of Biology, Dyson College of Arts and Sciences Pace University New York New York USA; ^12^ Present address: Department of Environmental Sciences, Zoology University of Basel Basel Switzerland; ^13^ Present address: Department of Biology University of Massachusetts Boston Boston Massachusetts USA; ^14^ Present address: School of Environment and Science Griffith University Southport Queensland Australia; ^15^ Present address: Department of Aquaculture and Fish Biology Hólar University Hólar Iceland; ^16^ Present address: Department of Computational Biology Cornell University Ithaca New York USA; ^17^ Present address: Wildlife Research Division Environment and Climate Change Canada Saskatoon Saskatchewan Canada; ^18^ Present address: School of Agriculture, Food and Ecosystem Sciences The University of Melbourne Melbourne Victoria Australia; ^19^ Present address: College of Fisheries and Ocean Sciences University of Alaska Fairbanks Juneau Alaska USA; ^20^ Present address: Fisheries, Aquatic Science, and Technology (FAST) Laboratory Alaska Pacific University Anchorage Alaska USA; ^21^ Present address: College of Fisheries and Ocean Sciences University of Alaska Fairbanks Fairbanks Alaska USA; ^22^ Present address: Alaska Ocean Observing System Anchorage Alaska USA

**Keywords:** contemporary evolution, darwins, haldanes, phenotypic change, phenotypic evolution, quantitative traits, time series

## Abstract

Populations must continuously respond to environmental change or risk extinction. These responses can be measured as phenotypic rates of change, which allow researchers to predict their contemporary evolutionary responses. In 1999, a database of phenotypic rates of change in wild populations was compiled. Since then, researchers have used (and expanded) this database to examine the phenotypic responses as a function of the features of the study system (i.e., the population or set of populations, of a given species, that experienced a specific driver or disturbance), the measured traits, and methodological approaches. Therefore, PROCEED (Phenotypic Rates of Change Evolutionary and Ecological Database) is an ongoing compilation of rates of phenotypic change, typically calculated as Haldanes and Darwins, published in peer‐reviewed literature (but also including data from theses and technical reports). Studies in this database measure the intraspecific change in quantitative (continuous or discrete) traits and report either the time elapsed from the onset of environmental novelty, or reference a historical or biological event reported in other sources (e.g., a mine opening or a well‐documented biological invasion). Included studies either follow a single population through time (allochronic design) or compare two or more populations that diverged at a known time (synchronic design). Some included studies account for the total phenotypic variability in the field (i.e., phenotypic studies), while others employed common‐garden or other quantitative genetic approaches to account for the heritable component of the phenotypic change (i.e., genetic studies). PROCEED includes systems in both natural and experimental conditions, provided that reproduction was not manipulated (i.e., artificial selection experiments were excluded). In the included experimental systems, the environment of the focal populations was manipulated (e.g., an herbivory exclusion experiment, where the type and load of herbivory are manipulated) but the studies did not deliberately select for trait values in the study population (e.g., the plant height). PROCEED does not include systems where the phenotypic change is presumably due to interspecific hybridization, polyploidy, or other chromosomal alterations. Here, we present the most recently updated PROCEED (Version 6.1). This new, curated version has 9263 records (*n*) collated from 326 studies, 1801 systems, and 428 species. The database includes records belonging to mammals (*n* = 686), birds (*n* = 1475), reptiles (*n* = 96), amphibians (*n* = 23), fishes (*n* = 3671), invertebrates (*n* = 1141, mostly arthropods), and plants (*n* = 2171). The maximum elapsed time between the environmental change and the sampling is 500 years but is typically less than 100 years (third quartile 89.5; median 45 years). The database also includes a set of variables describing biological and methodological aspects of the study system and measured traits, along with features of the sampling design in the primary source of information. This new version of PROCEED also includes a time series dataset comprising a subset of records included in the general dataset. These are allochronic studies with three or more sampling times throughout the entire study period. The time series dataset contains 655 time series (*s*)—belonging to 61 studies, from 156 systems, and 77 species—including mammals (*s* = 140), birds (*s* = 77), reptiles (*s* = 4), amphibians (*s* = 8), fishes (*s* = 404), and plants (*s* = 22). The data are released under a Creative Commons CC0 1.0 Universal Public Domain Dedication license.

## CONFLICT OF INTEREST STATEMENT

The authors declare no conflicts of interest.

## Supporting information


Appendix S1.

## Data Availability

The complete data set is available as Supporting Information and is also available in Borealis (The Canadian Dataverse Repository) at https://doi.org/10.5683/SP3/NXSL3Q.

