# Supplementary material for: PROCEED v6.1: Phenotypic rates of change evolutionary and ecological database
Source: Ecology. 2025 Mar 3;106(3):e70009. doi: 10.1002/ecy.70009 (PMC11876790; doi:10.1002/ecy.70009)
Supplement: Supplementary file 1 — Appendix S1. [file ECY-106-e70009-s001.zip › Metadata_S1.pdf]

Data Paper published in *Ecology*, an Ecological Society of America journal

Metadata S1

Title:

PROCEED v6.1: Phenotypic rates of change evolutionary and ecological database

Authors:

Lucas D. Gorné; Andrew P. Hendry; Fanie Pelletier; Sarah Sanderson; Cristian Correa Carlos F. Arias; Marc-Olivier Beausoleil; Maryse Boisjoly; Erika Crispo; Daniel Berner; Luis F. De León; Joseph D. DiBattista; Grant E. Haines; Benjamin C. Haller; Michael T. Kinnison; Shahin Muttalib; Ann E. McKellar; Rose E. O'Dea; Winer Daniel Reyes-Corral; Yanny Ritchot; Krista B. Oke; Zachary T. Wood; Thomas Farrugia; Kiyoko M. Gotanda

Open Research statement:

The complete data set is available as Supporting Information and is also available in Borealis (The Canadian Dataverse Repository) at https://doi.org/10.5683/SP3/NXSL3Q.

Introduction

The Phenotypic Rates of Change Evolutionary and Ecological Database (PROCEED) is an ongoing compilation of rates of phenotypic change. PROCEED provides information to compute two popular and complementary evolutionary rates, Darwins and Haldanes, in contemporary timescales. PROCEED also provides a set of biological and methodological variables aimed at modeling and understanding the variability in those rates of phenotypic change.

The Darwins (*D*) rate of change is the proportional change in a quantitative trait (*x*) over time (Haldane 1949).

$$D = \frac{1}{x} \cdot \frac{dx}{dt}$$

The integral of this equation across a discrete period ($t=t_2-t_1$) is the difference between the natural logarithm of a trait value at a particular time (or in a particular population) and the natural logarithm of the same trait at some time in the past (or in another population), over the length of time (typically expressed as millions of years).

$$D = \frac{\ln(\bar{x}_2) - \ln(\bar{x}_1)}{t_2 - t_1}$$

$$D = \frac{\ln(\bar{x}_2 / \bar{x}_1)}{t}$$

This means that the value of the trait at time 2 is a function of the value at time 1 as follows:

$$\bar{x}_2 = \bar{x}_1 \cdot e^{D \cdot t}$$

The Haldanes (*H*) rate of change (Haldane 1949) scales the magnitude of change by the amount of variation in the trait. Lerman (1965) further articulated this measure by expressing rates as "differences

between the population means in units of standard deviation." Gingerich (1993) formulated a similar metric and dubbed it the Haldane (the same measure is designated D' H by Lynch 1990). A simple formulation of the Haldane is:

$$H = \frac{\frac{\bar{x}_2 - \bar{x}_1}{SD_x}}{g}$$

where x_2 and x_1, represent mean trait values for each of two populations (synchronic) or for a single population at two different times (allochronic), SD_x is the pooled standard deviation, and g is the number of generations separating the populations or samples (years divided by generation time). For many traits, especially morphological traits, standard deviations are expected to increase with the mean (i.e., coefficient of variation remains relatively constant). For such traits, raw data should be transformed to natural logarithms (ln), which will reduce heteroscedasticity (Wright 1968). However, estimated rates of change calculated using ln means and standard deviations do not differ appreciably from those calculated using non-transformed means and standard deviations (Hendry & Kinnison 1999).

Darwins and Haldanes differ in two fundamental ways. First, Darwins specify the rate of proportional change in units of the initial value assuming an exponential rate of change, whereas Haldanes specify the rate of change in standard deviation units. Second, the time interval is measured per millions of years for Darwins and in generations for Haldanes. In principle (but not in practice), these two differences are independent of each other. Indeed, a proportional change could just as easily be specified per generation or a standardized change per year. If an investigator's goal is to measure a change in some organism that is relevant to time dependent human interests, then proportionate change and a time unit based on years could be the most desirable characteristic of a rate measure (i.e., Darwins). However, if the

goal is to understand how a population responds to environmental change or to estimate the intensity of selection, then standardizing by the trait's variation and using a time scale more relevant to the organism (i.e., generations) will provide a more appropriate rate measure (i.e., Haldanes).

In accounting for variation, Haldanes have a better grounding in the evolutionary process, but this property adds additional complexity when comparing genetic and phenotypic studies. Environmentally-induced variation might be greater in the wild than it is under common conditions. In such cases, genetic Haldanes will be higher than phenotypic Haldanes for the same absolute magnitude of change. In contrast, if individuals with extreme phenotypes are selected against in the wild, but not in the laboratory, genetic Haldanes may be lower than phenotypic Haldanes for the same magnitude of change.

The accuracy of an estimated evolutionary rate depends on the accuracy of estimated elapsed time (years for Darwins, generations for Haldanes). Estimating the number of years that have passed might seem easy, but it is not without ambiguity in many instances. Small errors in the estimated year of population founding, for example, can translate into measurable differences in evolutionary rates when the total time interval is relatively short. Obtaining good estimates of the time interval in generations is often more difficult because of the added uncertainty in the estimation of generation time. As a result, rates estimated in Haldanes may be less accurate than those estimated in Darwins, regardless of how well each measure reflects the evolutionary process.

The dimension in which a trait is measured (e.g., length, area, volume) influences rates of evolution calculated using Darwins (Gingerich 1993). This dimension dependence arises because as length increases, area increases as the square function of that change, and volume as the cube function of that

change (assuming a cubic trait). On the other hand, Haldanes are not dimension dependent. Phenotypic data is commonly measured on ratio or interval scales. Data on a ratio scale have a constant interval between adjacent units, and the measurement scale has a precise zero point corresponding to a null quantity. Ratio scale data have the property that doubling a value doubles the actual quantity. In contrast, data on an interval scale have a constant interval between adjacent units, but the zero point is arbitrary (e.g., time of day). Darwins are not appropriate for specifying rates of evolution for interval scale traits. Behavioral and phenological traits are often measured on an interval scale, such as migratory timing, or flowering time. Darwins cannot be used to estimate rates in those cases because, for example, in the migration case, *What is day zero?* If it is assumed to be the first day of the year, the rate of change will be different from the rate of change for the same amount of change if day zero is assumed to be June 1. On the contrary, Haldanes is still suitable for calculating evolutionary rates on interval scales.

In a similar way to the issues with the interval scales, for morphological traits, the choice of the landmarks may impact calculated Darwins if an evolutionary change is not proportional along its entire structure being measured (i.e., the change occurs in a smaller section of the structure). In those cases, the landmark operates as a misleading zero. This discrepancy arises because the same change in only part of the structure will appear smaller when measured from a more distant zero point. Haldanes are not as strongly influenced as Darwins by the choice of different landmark points when part of the trait is invariant. Furthermore, both Darwins and Haldanes are ill-equipped to handle rates of change in nonmetric characters, such as color morphs, presence or absence of a character state, behavioral options, or alternative life histories.

Darwins and Haldanes provide different but complementary measures of evolutionary rate. Because both specify a phenotypic difference per unit time, rates calculated using each will be correlated in general. However, some studies might have relatively low Haldanes rates but high Darwins rates, or the opposite, because of differences in the time units (years vs generations), or because of the unit of change (trait initial value vs trait variance). Due to the different conceptual basis of the two measures and to the different information conveyed by each, it is worth presenting both when possible. It is also important to acknowledge the limitations of each measure as discussed above.

PROCEED includes studies that measure the intraspecific change in quantitative (continuous or discrete) traits and report the time elapsed from the onset of environmental novelty or a reference to a historical or biological event reported in other sources (e.g., a mine opening, a well-documented biological invasion). The maximum elapsed time between the environmental change and the sampling was no longer than 500 years (figure 1). However, most of the observations correspond to less than 100 years of elapsed time (3rd quartile 89.5 years) and only a few cases correspond to more than 200 years of elapsed time (figure 1). The time measured as years and generations were positively correlated, but for a given time in years, there is a wide range of variability in generations, approximately two orders of magnitude (figure 1). The included studies followed a single population through time (allochronic design) or compare two or (more) populations that diverged at a known time (synchronic design). The studies may record the differences among populations in the field (here called phenotypic studies) or in common growing conditions (here called genetic studies). The database records the change in diverse trait types and environmental changes (Table 1, see “Variable information” section for definitions).

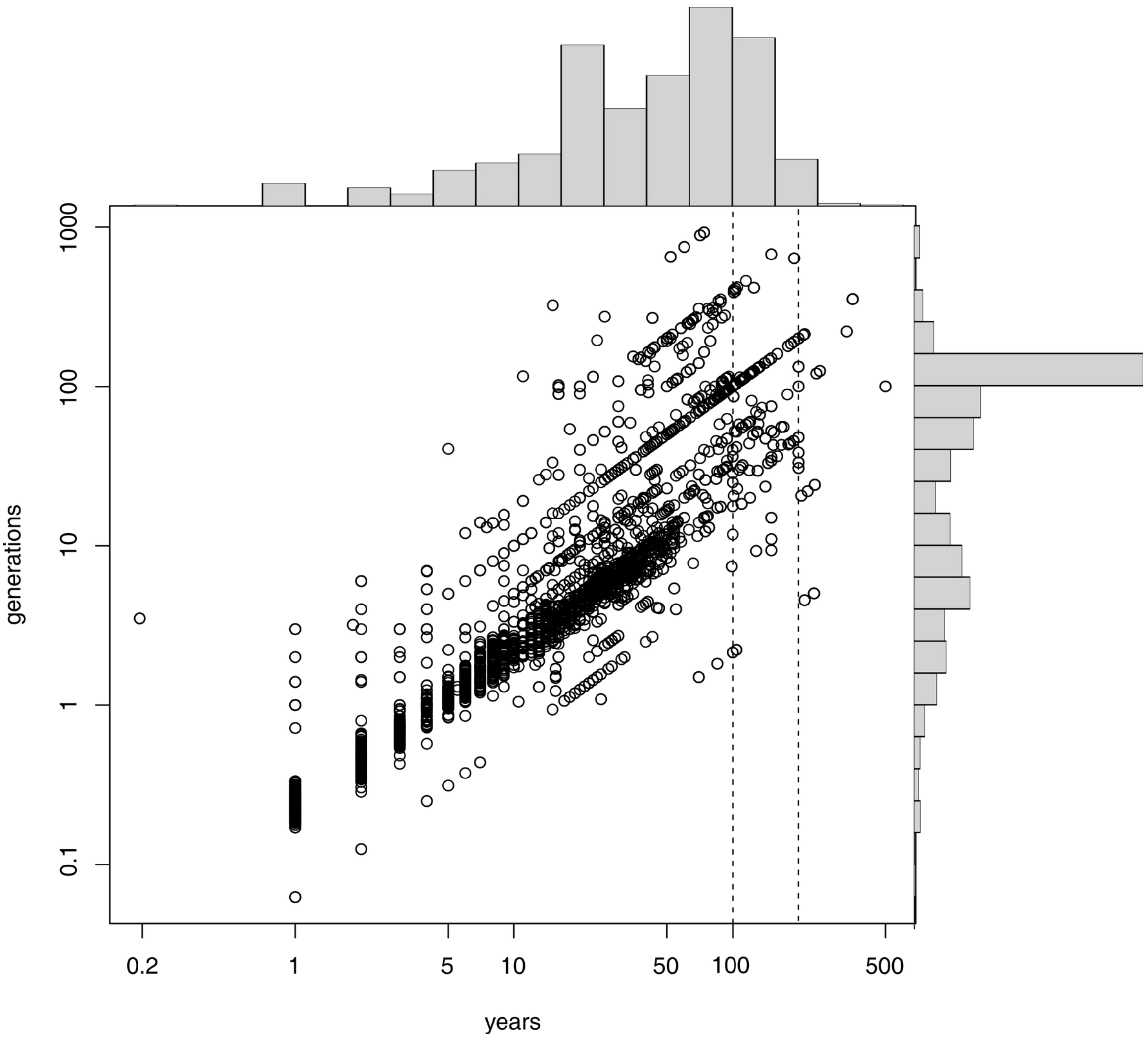

Figure 1. *Distribution of elapsed times in PROCEED both, in years and in generations on a log scale. The vertical dashed lines point out 100 and 200 years respectively.*

In 1999, a database of phenotypic rates of change in wild populations was compiled. Since then, researchers have used (and expanded) this database to examine the phenotypic responses as a function of the features of the study system (i.e., the population or set of populations, of a given species, that experienced a specific driver or disturbance), the measured traits, and methodological approaches. We

compile and add data regularly to the dataset. This dataset is continually being updated as more people include new variables and observations. PROCEED v6.1 is now being released. This last version has 9263 observations, from 1801 worldwide distributed systems (i.e., a population or a set of populations, of a given species, experiencing a specific driver/disturbance) (figure 2), belonging to 428 species. This information was extracted from 326 primary sources of information (i.e. papers, technical reports, etc.). Table 1 shows the distribution of the data in a selection of the classification factors in PROCEEDv6.1_RatesDB. For a full description of the variables and levels see the "Variable information" section.

Table 1. *Distribution of observations, systems, and species by a selection of classification factors in PROCEEDv6.1_RatesDB.*

Factor<br>level	Observations	Systems	Species
Taxa			
Amphibian	23	5	5
Annelid	1	1	1
Arthropod	1095	20	17
Bird	1475	201	172
Fish	3671	1311	44
Mammal	686	98	63
Mollusc	45	4	4
Plant	2171	156	118
Reptile	96	5	4
design			
Allochronic	3435	1598	283
Synchronic	5828	203	145
genphen			
Genetic	3063	104	75
Phenotypic	6200	1697	353
trait_type			
behaviour	19	3	3
growth	332	17	12
otherLH	1518	59	34
othermorphology	3059	117	68
phenology	164	100	82
physio	634	37	24
response	78	14	12
size	3459	1454	193
disturbance			
Climate change	451	231	182
Hunt_harv	1365	794	31
Introduction	4979	129	88
Landscape change	375	38	25
Other	1529	551	55
Pollution	216	35	25
Response to introductions	348	23	22

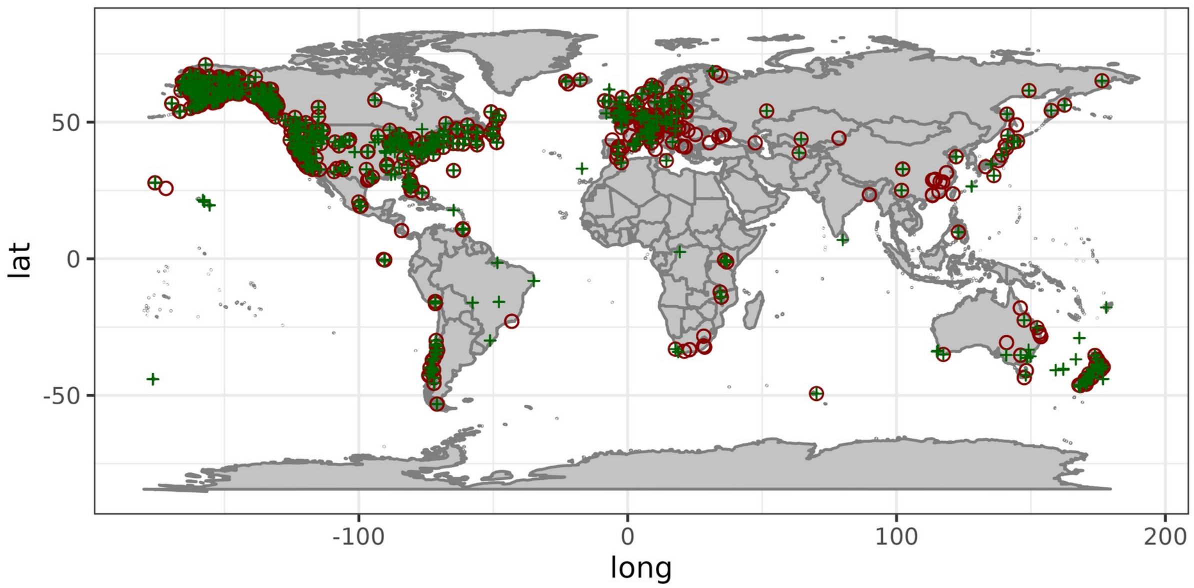

Figure 2. *Geographic distribution of the populations recorded in PROCEED. Red circles show the position of the sample 1 (original population). Green crosses show the position of the sample 2 (derivative population).*

Figure 3 shows the joint distribution of Darwins and Haldanes in PROCEED. According to previous definitions and considerations, Darwins cannot be computed if the trait is not measured in a ratio scale, or if the average mean of sample 1 (in a raw scale) is equal to zero, or if it is a missing value, or if the ratio between mean 2 and mean 1 (in a raw scale) is a negative value (414 records were excluded). Additionally, we did not compute Darwins if the trait measurement scale is unknown whether it is in the raw scale or any transformation (e.g. logarithmic) was applied. Only data in the raw or ln-transformed scale were included (89 additional records were excluded). Here, Darwins numerators were computed in the raw scale of the trait. If the scale of the trait was ln-transformed (ln: natural logarithm), the Darwins numerator was computed according to the Lynch (1990) approximation. That is, the natural logarithm of

the average of the trait at a given time is approximated as the average of the natural logarithm of the trait plus the half of the variance of the natural logarithm of the trait:

$$\ln(\bar{x}_t) = \overline{\ln(x_t)} + 0.5 \cdot \sigma^2_{\ln(x_t)}$$

As a consequence, the records expressed in ln-transformed scale also requires the standard deviation to be computed. Thus, 202 additional records were excluded. Additionally, four records were assumed to be outliers and excluded because the ratio between the mean in sample 1 and sample 2 was larger than two orders of magnitude.

According to previous definitions and considerations, Haldanes cannot be computed if the standard deviation of sample 1 or 2, or any of the sample sizes are lacking (1620 records were excluded). Also, it cannot be computed if the generation time is unknown (274 additional records were excluded). Additionally, we assumed as outlier and excluded from the calculation of Haldanes any records where the standard deviation of any of the samples were zero or the coefficient of variation was smaller than 0.1 or larger than 1000 (79 additional records were excluded).

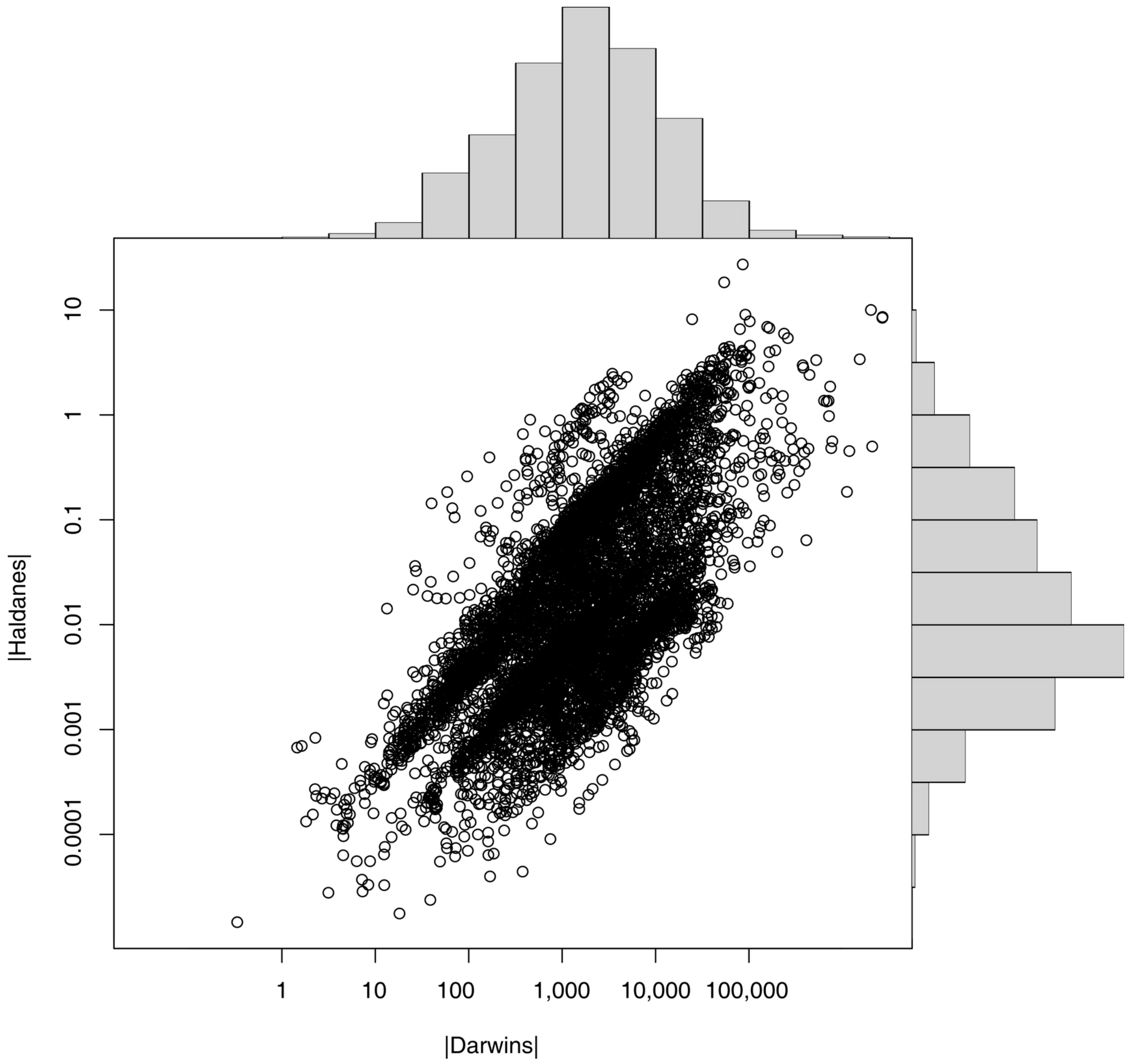

Figure 3. *Distribution of absolute values for Darwins and Haldanes in PROCEED.*

The dataset PROCEEDv6.1_TimeSeriesDB unpacks a subset of allochronic observations from the main dataframe. The time-series dataset compiles 655 time-series from 156 systems and 77 species. Table 2 summarizes the distribution of the series in a selection of the classification factors. Figure 4 shows the frequency distribution of the time-series length measured as both years and time-points. When the driver

of change in a population was modified, the new situation was classified as a new system. The new system is linked to the previous situation via “released_sys”. For example, the system s445 (*Ovis canadensis* in Ram Mountain, Alberta, Canada when a hunting moratorium was placed starting in 1996) is the same population of system s122 (*Ovis canadensis* in Ram Mountain, Alberta, Canada when hunting was allowed up to 1995). So, for the system s445 “released_sys”=”s122”. On the contrary, for the system s122 “released_sys”=NA (because s122 was not derived from any other system in the database). Because of this criterion, nine cases produced time series with only 2 time-points (belonging to 6 systems and 2 fish species). This is an exception to the general rule of including series with three or more time-points.

Table 2. *Distribution of time-series, systems, and species by a selection of classification factors in PROCEEDv6.1_TimeSeriesDB.*

Factor<br>level	Series	Systems	Species
Taxa			
Amphibian	8	2	2
Bird	77	25	15
Fish	404	58	20
Mammal	140	58	27
Plant	22	12	12
Reptile	4	1	1
genphen			
Genetic	2	1	1
Phenotypic	653	155	76
trait_type			
growth	59	9	2
otherLH	78	10	8
othermorphology	176	63	32
phenology	22	15	6
physio	10	6	6
size	310	53	23
disturbance			
Climate change	132	68	30
Hunt_harv	322	48	19
Introduction	9	4	3
Landscape change	8	5	5
Other	171	24	13
Pollution	8	6	6
Response to introductions	5	1	1

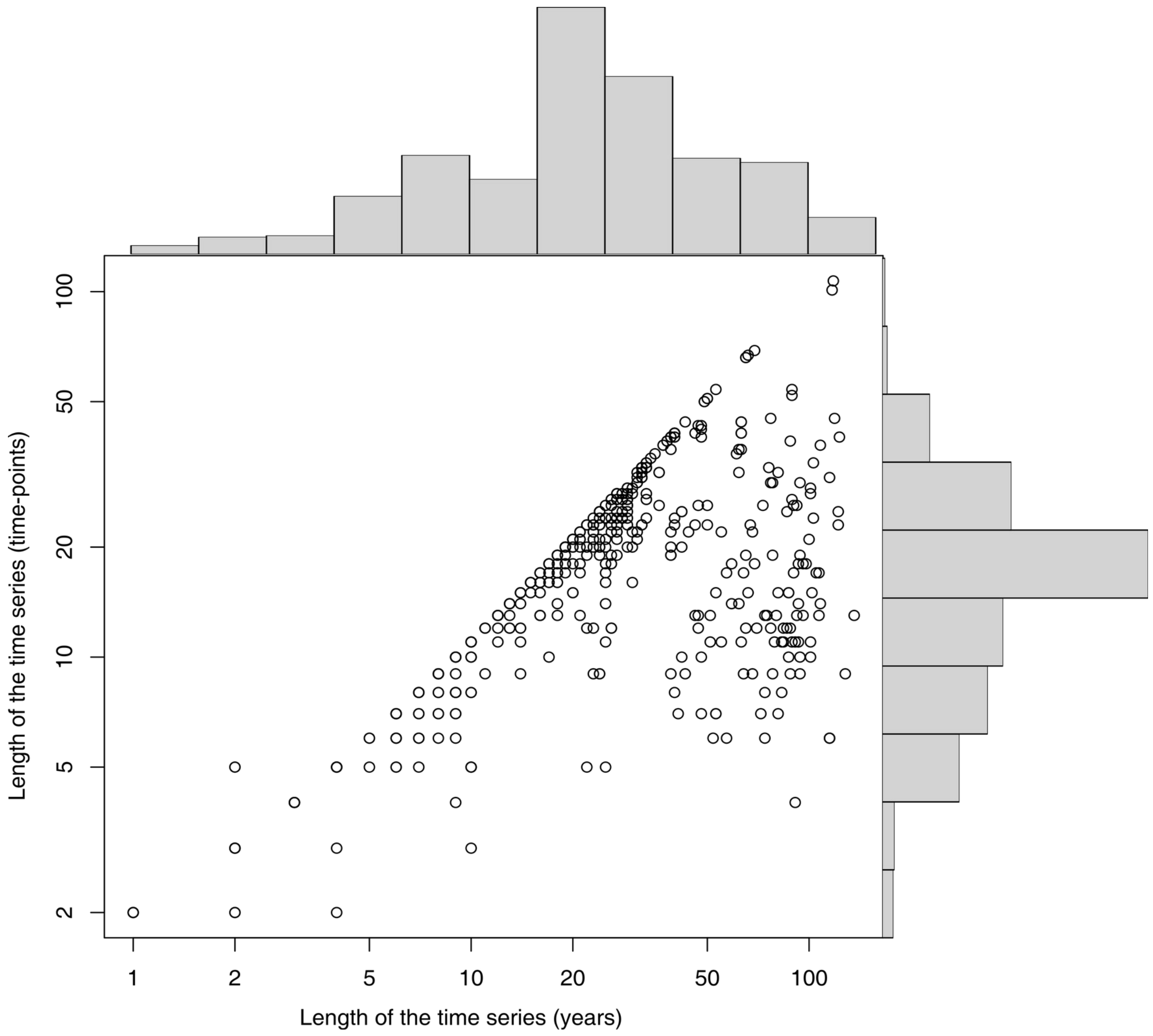

Figure 4. *Length of the time series in PROCEED, showed both, in years, and in time points.*

The datasets PROCEEDv6.1_species_genome and PROCEEDv6.1_species_GT are complementary tables with additional information about the genome size and generation time respectively of the species in the Rates and TimeSeries datasets (Figure 5). Rates, TimeSeries, and taxonomy tables are structured as a relationship database linked by primary and foreign keys (Figure 5). The complementary tables

summarize information collected for each species and inform specific attributes of Rates and TimeSeries according to the criteria explained in the metadata.

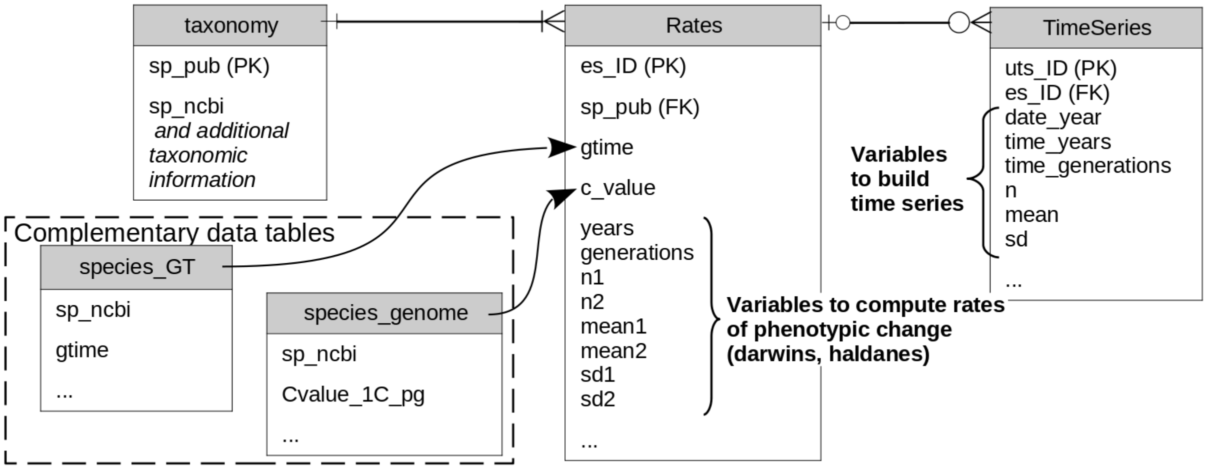

Figure 5. *Entity Relationship Diagram of PROCEED. "Rates" inherits sp_ncbi and additional taxonomic information from the"taxonomy" entity. For each element in "taxonomy" there are from one to many elements in "Rates". For each element in "Rates" there is one element in "taxonomy". "TimeSeries" inherits attributes from "Rates". For each element in "Rates" there might be from zero to many elements in "TimeSeries". For each element in "TimeSeries", there might be from zero to one element in "Rates". Among the "Rates" attributes, are those required to compute Darwins and Haldanes. Among the attributes in "TimeSeries", are those required to build the time series of phenotypic change in a study system. "..." denotes there are more attributes not listed here. See the metadata of each dataset for further details. PK: primary key; FK: foreign key. The complementary tables inform specific attributes of the "Rates" entity. "species_GT" and "species_genome" summarize all the generation time and genome size information collected for each species (sp_ncbi) respectively but there is not a PK for these two tables because more than one generation time or genome size can be recorded for each species.*

PROCEED has been compiled to answer several questions about how phenotypic evolution is displayed in contemporary time scales and how different factors affect this process (its pattern, rate, and amount of change). Some of these factors have been introduced previously (factors listed in Table 1, for example) and have been analyzed with previous versions of PROCEED. Since its early versions, PROCEED has been the empirical support for a number of eco-evolutionary studies (Hendry & Kinnison 1999; Kinnison & Hendry 2001; Hendry et al. 2008; Darimont et al. 2009; Crispo et al. 2010; Westley 2011; Palkovacs et al. 2012; Gotanda et al. 2015; Alberti et al. 2017; Gorné & Díaz 2019; Sanderson et al. 2021, 2023). Some of these papers are fundamental contributions to the field of ecology and evolution. For example PROCEED showed that phenotypic change in the contemporary timescales is consistent with an abrupt initial change followed by a period of stasis or some degree of random changes (Kinnison & Hendry 2001; Gorné & Díaz 2019). The database also showed that the most rapid phenotypic changes tend to be in populations facing human disturbances such as pollution, urbanization, landscape change, invasive species, or hunting/harvesting (Alberti et al. 2017; Westley 2011; Sanderson et al. 2021; Darimont et al. 2009). In particular, pollution and hunting/harvesting by humans caused more rapid changes compared to populations experiencing other types of disturbances (Darimont et al. 2009; Sanderson et al. 2021), and populations in urbanized systems have faster rates of change than populations in non-urbanized systems (Alberti et al. 2017). PROCEED also showed that the rates of change in life history traits are as fast or even faster than the rates of change in morphological traits (Kinnison & Hendry 2001; Gorné & Díaz 2019). The new version of PROCEED would allow us to dig deeper into human influences on rates of trait change as well as to address different questions like the relationship between some biological properties of the species (e.g. generation time, genome size, body size) and the patterns of phenotypic

change. Future work can also look at different combinations of things known to affect trait change to see if they have synergistic or antagonistic effects on phenotypic change.

Here, as an example, we performed a similar analysis to the one by Sanderson et al (2021) to inquire about the effect of different human influences on the amount of phenotypic change they drive.

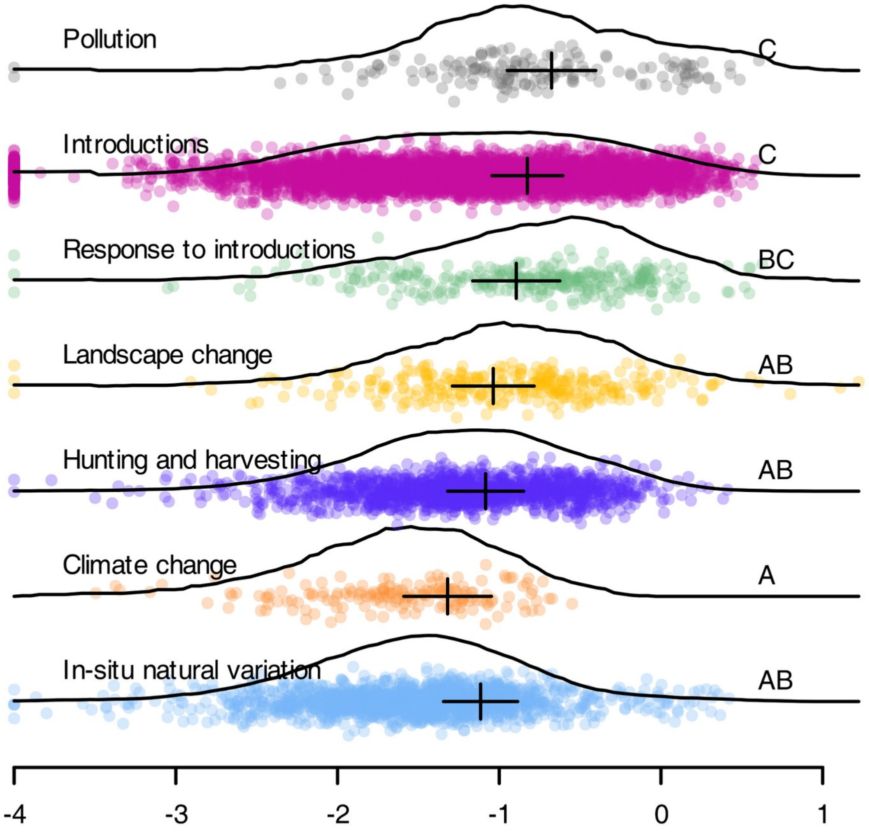

Figure 6. *Amount of phenotypic change—in log_{10}-transformed absolute darwin numerators—for six types of disturbances (pollution, response to introductions, hunting and harvesting, introductions,*

landscape change, climate change) and in situ natural variation (disturbance=”Other”). Points show individual data, lines show smoothed data distributions, and crosses show linear mixed model estimates and 95% confidence interval for each disturbance type taking into account all the other variables in the database that might affect the observed amount of change. Letters indicate groups that differ according to Tukey's tests based on the fitted model.

As previously reported by Sanderson et al (2021), we found that systems associated with pollution have the highest amounts of change and that systems associated with climate change have the lowest change (Figure 6). High rates of change due to pollution might be explained by the underlying genetic architecture of tolerance to pollutants (Macnair 1991; Oomen et al. 2020; Kardos & Luikart 2021). The R code to reproduce Figure 6, as well as code for some basic handling of PROCEED and rates computing, is available in the file “PROCEEDv6.1_BasicCode.txt”.

PROCEEDv6.1_BasicCode has R code for some basic handling of PROCEEDv6.1 and examples to perform some basic analyses and graphics. See the metadata of each dataset for further details. Additionally to the stored PROCEED v6.1 in the stable repository (https://doi.org/10.5683/SP3/NXSL3Q), previous versions are available in the webpage of the database (https://proceeddatabase.weebly.com/) as well as in GitHub (https://github.com/photopidge/PROCEED).

Class I. Data Set Descriptors

A Data set identity: PROCEED v6.1: Phenotypic rates of change evolutionary and ecological database

B Data set identification code:

PROCEEDv6.1_RatesDB.csv

PROCEEDv6.1_TimeSeriesDB.csv

PROCEEDv6.1_species_genome.csv

PROCEEDv6.1_taxonomy.csv

PROCEEDv6.1_species_GT.csv

PROCEEDv6.1_BasicCode.txt

C. Data set description

1. Originators:

Lucas D. Gorné (Córdoba, Argentina); Andrew P. Hendry (Montreal, Canada); Fanie Pelletier (Sherbrook, Canada); Sarah Sanderson (Montreal, Canada); Cristian Correa (Valdivia, Chile); Michael T. Kinnison (Orono, ME, USA); Kiyoko M. Gotanda (St. Catharines, Canada).

2. Abstract:

Populations must continuously respond to environmental change or risk extinction. These responses can be measured as phenotypic rates of change, which allow researchers to predict their contemporary evolutionary responses. In 1999, a database of phenotypic rates of change in wild populations was compiled. Since then, researchers have used (and expanded) this database to examine the phenotypic responses as a function of the features of the study system (i.e., the

population or set of populations, of a given species, that experienced a specific driver or disturbance), the measured traits, and methodological approaches. Therefore, PROCEED (Phenotypic Rates of Change Evolutionary and Ecological Database) is an ongoing compilation of rates of phenotypic change, typically calculated as Haldanes and Darwins, published in peer-reviewed literature (but also including data from theses and technical reports). Studies in this database measure the intraspecific change in quantitative (continuous or discrete) traits and report either the time elapsed from the onset of environmental novelty, or reference a historical or biological event reported in other sources (e.g., a mine opening or a well-documented biological invasion). Included studies either follow a single population through time (allochronic design) or compare two or more populations that diverged at a known time (synchronic design). Some included studies account for the total phenotypic variability in the field (i.e., phenotypic studies), while others employed common-garden or other quantitative genetic approaches to account for the heritable component of the phenotypic change (i.e., genetic studies). PROCEED includes systems in both natural and experimental conditions, provided that reproduction was not manipulated (i.e., artificial selection experiments were excluded). In the included experimental systems, the environment of the focal populations was manipulated (e.g., an herbivory exclusion experiment, where the type and load of herbivory are manipulated) but the studies did not deliberately select for trait values in the study population (e.g., the plant height). PROCEED does not include systems where the phenotypic change is presumably due to interspecific hybridization,

polyploidy, or other chromosomal alterations. Here, we present the most recently updated PROCEED (Version 6.1). This new, curated version has 9263 records (n) collated from 326 studies, 1801 systems, and 428 species. The database includes records belonging to mammals (n=686), birds (n=1475), reptiles (n=96), amphibians (n=23), fishes (n=3671), invertebrates (n=1141, mostly arthropods), and plants (n=2171). The maximum elapsed time between the environmental change and the sampling is 500 years but is typically less than 100 years (3rd quartile 89.5; median 45 years). The database also includes a set of variables describing biological and methodological aspects of the study system and measured traits, along with features of the sampling design in the primary source of information. This new version of PROCEED also includes a time-series dataset comprising a subset of records included in the general dataset. These are allochronic studies with three or more sampling times throughout the entire study period. The time-series dataset contains 655 time-series (s)—belonging to 61 studies, from 156 systems, and 77 species—including mammals (s=140), birds (s=77), reptiles (s=4), amphibians (s=8), fishes (s=404), and plants (s=22).The data are released under a Creative Commons CC0 1.0 Universal Public Domain Dedication license.

D. Key words/phrases:

Contemporary evolution, darwins, haldanes, phenotypic change, phenotypic evolution, quantitative traits, time series.

Class II. Research origin descriptors

A Overall project description:

PROCEED is an ongoing compilation of rates of phenotypic change, typically Haldanes and Darwins, published in peer-reviewed manuscripts (but also including data from theses and technical reports). It aims to analyze the tempo and mode of phenotypic change in contemporary time scales and the factors affecting these patterns and rates.

A.1 Identity:

Phenotypic rates of change evolutionary and ecological database (PROCEED)

A.2 Originators:

Andrew P. Hendry (Montreal, Canada); Fanie Pelletier (Sherbrook, Canada); Michael T. Kinnison (Orono, ME, USA); Kiyoko M. Gotanda (St. Catharines, Canada).

A.3 Period of study:

1999 to 2024.

A.4 Objectives:

To analyze the tempo and mode of phenotypic change in contemporary time scales and the factors affecting these patterns and rates.

A.5 Abstract:

Populations must continuously respond to environmental change or risk extinction. These responses can be measured as phenotypic rates of

change, which allow researchers to predict their contemporary evolutionary responses. In 1999, a database of phenotypic rates of change in wild populations was compiled. Since then, researchers have used (and expanded) this database to examine the phenotypic responses as a function of the features of the study system (i.e., the population or set of populations, of a given species, that experienced a specific driver or disturbance), the measured traits, and methodological approaches. Therefore, PROCEED (Phenotypic Rates of Change Evolutionary and Ecological Database) is an ongoing compilation of rates of phenotypic change, typically calculated as Haldanes and Darwins, published in peer-reviewed literature (but also including data from theses and technical reports). Studies in this database measure the intraspecific change in quantitative (continuous or discrete) traits and report either the time elapsed from the onset of environmental novelty, or reference a historical or biological event reported in other sources (e.g., a mine opening or a well-documented biological invasion). Included studies either follow a single population through time (allochronic design) or compare two or more populations that diverged at a known time (synchronic design). Some included studies account for the total phenotypic variability in the field (i.e., phenotypic studies), while others employed common-garden or other quantitative genetic approaches to account for the heritable

component of the phenotypic change (i.e., genetic studies). PROCEED includes systems in both natural and experimental conditions, provided that reproduction was not manipulated (i.e., artificial selection experiments were excluded). In the included experimental systems, the environment of the focal populations was manipulated (e.g., an herbivory exclusion experiment, where the type and load of herbivory are manipulated) but the studies did not deliberately select for trait values in the study population (e.g., the plant height). PROCEED does not include systems where the phenotypic change is presumably due to interspecific hybridization, polyploidy, or other chromosomal alterations. Here, we present the most recently updated PROCEED (Version 6.1). This new, curated version has 9263 records (n) collated from 326 studies, 1801 systems, and 428 species. The database includes records belonging to mammals (n=686), birds (n=1475), reptiles (n=96), amphibians (n=23), fishes (n=3671), invertebrates (n=1141, mostly arthropods), and plants (n=2171). The maximum elapsed time between the environmental change and the sampling is 500 years but is typically less than 100 years (3rd quartile 89.5; median 45 years). The database also includes a set of variables describing biological and methodological aspects of the study system and measured traits, along with features of the sampling design in the primary source of information. This new version of PROCEED also

includes a time-series dataset comprising a subset of records included in the general dataset. These are allochronic studies with three or more sampling times throughout the entire study period. The time-series dataset contains 655 time-series (s)—belonging to 61 studies, from 156 systems, and 77 species—including mammals (s=140), birds (s=77), reptiles (s=4), amphibians (s=8), fishes (s=404), and plants (s=22).

A.6Sources of funding:

- Natural Sciences and Engineering Research Council of Canada (NSERC)
- Brock University
- McGill University
- Le Fonds Québécois de la Recherche sur la Nature et les Technologies (FQRNT)
- National Science Foundation

Class III. Data set status and accessibility

A Status

A.1 Latest update:

June 2024

A.2 Latest archive date:

June 2024

A.3 Metadata status:

June 2024

A.4 Data verification:

Data quality assurance checking finished.

B Accessibility

B.1 Storage location and medium:

https://doi.org/10.5683/SP3/NXSL3Q.

B.2 Contact persons:

Kiyoko M. Gotanda; Brock University, Department of Biological Sciences, 1812 Sir Isaac Brock Way, St. Catharines, ON L2S 3A1, Canada; kgotanda@brocku.ca

B.3 Copyright restrictions:

None. Creative Commons CC0 1.0 Universal Public Domain Dedication.

B.4 Proprietary restrictions:

None. Creative Commons CC0 1.0 Universal Public Domain Dedication.

Please, let Kiyoko M. Gotanda (kgotanda@brocku.ca) know when using PROCEED.

a. Release date: There are no restrictions.

b. Citation: Please cite both this data paper in *Ecology* and the dataset below:

Gotanda, Kiyoko M.; Gorné, Lucas D. (2024) "Phenotypic Rates of Change Evolutionary and Ecological Database (PROCEED)", version 6.1, Borealis, https://doi.org/10.5683/SP3/NXSL3Q.

b. Disclaimer(s): There are no disclaimers.

5. Costs: There are no costs to access and use PROCEED.

Class IV. Data structural descriptors 1

A Data set file

A.1 Identity: "PROCEEDv6.1_RatedDB.csv"

A.2 Size: 9263 rows, 69 columns. 10.8 MB.

A.3 Format and storage mode: CSV file; Language = English; Decimal point = "."; Not available data (NA) = "" (i.e. empty cell).

A.4 Header information: "PROCEEDv6.1_RatedDB.csv" includes a header with variable names for each column. In section IV.B we describe each variable.

A.5 Alphanumeric attributes: Mixed.

A.6 Special characters/fields: We avoided the use of special characters and comments were placed in specific columns (variables).

A.7 Authentication procedures: There are no authentication procedures to access the data. It is freely available in the Borealis repository (https://doi.org/10.5683/SP3/NXSL3Q).

B Variable information (description by column)

1. es_ID: Unique identifier for each individual rate.

2. ref_ID: Unique identifier for each primary source of information (paper, thesis, report, etc.).

3. sys_ID: Unique identifier for each study system (see "system" for the definition).

4. released_sys: sys_ID of the system originating the new system after removal/extinguishment/stop of the driver/disturbance. For example, the system s445 (*Ovis canadensis* in Ram Mountain, Alberta, Canada when a hunting moratorium was placed starting in 1996) is the same population of system s122 (*Ovis canadensis* in Ram Mountain, Alberta, Canada when hunting was allowed up to 1995). So, for the system s445 “released_sys”=”s122”. On the contrary, for the system s122 “released_sys”=NA (because s122 was not derived from any other system in the database). Future studies with similar situations (i.e. a single population in periods with different evolutionary drivers) should include, in "released_sys" of the new condition, the sys_ID of the former condition.

5. released_es: es_ID of the case originating the new case after removal/extinguishment/stop of the driver/disturbance in the system. A system being released from the original driver generates a new system. If more than one trait is measured in both systems (the original and the new one), "released_es" links the cases in the new system with the measurements of the same trait in the original systems. For example, in the systems s122 and s445, two traits were measured: mean weight (kg) and mean horn length (cm). In s122, the change in

mean weight is the case “es_ID”=”es2798” and the change in mean horn length is the case “es_ID”=”es2799”. So, for the case es9277 (change in mean weight in the system s445), “released_es”=”es2798”. For the case es9278 (change in mean horn length in the system s445), “released_es”=”es2799”. On the contrary, for es2798 and es2799 (system s122), “released_es”=NA (because s122 was not derived from any other system in the database). Future studies with similar situations (i.e. a single population in periods with different evolutionary drivers) should include, in "released_es" of the new condition, the es_ID of the same traits in the former condition.

6. sp_pub: Species name (or subspecific taxon name) of the population(s) being assessed in each case, as was published in the primary source of information.

7. sp_ncbi: Species or subspecific taxon name of the population(s) being assessed in each case, according to the National Center for Biotechnology Information (https://www.ncbi.nlm.nih.gov/Taxonomy/Browser/wwwtax.cgi?mode=Root) in February 2022. Two species were not found in the NCBI database, *Stipa occidentalis* and *Diaptomus sanguineus*, these names were checked in The Plant List (http://www.theplantlist.org/) and WORMS (https://www.marinespecies.org/index.php) respectively. The supplementary table

"PROCEEDv6.1_taxonomy.csv" contains the complete list of "sp_pub" and its "sp_ncbi".

8. taxa: This is a coarse classification, not necessarily a clade (monophyletic group). Current categories are: Annelid, Mollusc, Arthropod, Fish, Amphibian, Mammal, Bird, Reptile, Plant.

9. phylum: Taxonomic clade of rank "phylum" including the studied species, according to the National Center for Biotechnology Information (https://www.ncbi.nlm.nih.gov/Taxonomy/Browser/wwwtax.cgi?mode=Root) last retrieved May 2022. The supplementary table "PROCEEDv6.1_taxonomy.csv" contains the complete list of "sp_ncbi" and its "phylum".

10. class: Taxonomic clade of rank "class" including the studied species, according to the National Center for Biotechnology Information (https://www.ncbi.nlm.nih.gov/Taxonomy/Browser/wwwtax.cgi?mode=Root) last retrieved May 2022. The supplementary table "PROCEEDv6.1_taxonomy.csv" contains the complete list of "sp_ncbi" and its "class".

11. order: Taxonomic clade of rank "order" including the studied species, according to the National Center for Biotechnology Information

(https://www.ncbi.nlm.nih.gov/Taxonomy/Browser/wwwtax.cgi?mode=Root) last retrieved May 2022. The supplementary table "PROCEEDv6.1_taxonomy.csv" contains the complete list of "sp_ncbi" and its "order".

12. system: It is a population or a set of populations, of a given species, experiencing a specific driver/disturbance. If the design is synchronic, the system includes both diverging populations (or sets of populations). Each system has its own unique combination of species, disturbance/driver and location. Within a given system, you can have multiple traits – for example, tarsus length and fledging date. We suggest keeping it general. Please note you can have both allochronic and synchronic data within the same system (e.g. Leaver 2012, Biol. J. Linnean. Soc. 107: 494-509). Example systems: "*Salmo trutta* (brown trout) monitored for 37 y following fish ladder construction (1966) in River Gudbrandsdalslågen, Norway"; "*Troglodytes troglodytes* (Eurasian Wren) migration arrival date monitored in its native range in Europe"; "*Oncorhynchus gorbuscha* (pink salmon) long term trends on northwest coast of US".

13. experimental: States if the phenotypic change is driven by experimentally set conditions (N=no; Y=yes). It is not artificial selection (i.e. selection of specific attributes by choosing breeders and manipulating mating) but a process of natural selection in an experimental environment.

14. exp_type: When "experimental"="N", "exp_type"=NA (empty cell). When "experimental"="Y", the following categories were applied:

- 01: A new environmental condition is imposed on a natural population, in the field, in situ.
- 03: A new environmental condition is imposed on a natural population, in the field, ex situ (transplant).
- 05: A new environmental condition is imposed on a natural population, in a common garden/greenhouse experiment.
- 07: A new environmental condition is imposed on an artificially generated population in the field. Here, the artificial population is a set of genotypes from the natural population, evenly represented by (full or half) sibling families.
- 09: A new environmental condition is imposed on an artificially generated population in a common garden experiment. Here, the artificial population is a set of genotypes from the natural population, evenly represented by (full or half) sibling families.
- 11: A new environmental condition is imposed on an artificial population (it was obtained by randomly crossing F2 individuals from a set of interbreeds between contrasting genotypes selected from a natural population) in a common garden experiment.

15. comments_on_system: Any comments about the system should be stated here.

16. c_value: A measurement of genome size, it is the amount (in picograms) of DNA contained within a haploid nucleus (e.g. a gamete) or one-half the amount in a diploid somatic cell. The genome size is a biological property of organisms that can affect a broad range of traits and biological processes (Gregory 2005), some of which could affect phenotypic change. Genome size is not related to the number of coding genes and most of the variability in genome size is due to noncoding DNA (Gregory 2001). However, noncoding DNA has genetic and epigenetic functions, as well as nongenetic effects that can affect or set conditions for the further evolution of organisms in different directions. At the intraspecific level, genome size is not consistently correlated with trait variability or plasticity (Meyerson et al. 2020). Some evidence shows that genome duplication increases the early response to selection (Martin & Husband 2012). On the contrary, some evidence points out that plant and vertebrate species (but not mammals) with large genomes are more prone to extinction (Vinogradov 2003; Vinogradov 2004; Knight et al. 2005). This could suggest that species with larger genome sizes have a lower ability to adapt.

In the context of rapid environmental changes, phenotypic plasticity and evolutionary responses based on genetic variance already existing in a population

can play a decisive adaptive role (Lande & Shannon 1996; Lacy 1997; Barrett & Schluter 2008; Stamp & Hadfield 2020). Genome size and mutation rate per locus are positively correlated (Sparrow et al. 1961; Abrahamson 1973), suggesting that higher genetic variance in larger genomes might be expected. Furthermore, due to the regulatory functions of noncoding DNA, we expect more phenotypic plasticity in large genome size species (Zuckerkandl 2002; Shanmugam et al. 2017). However, there is evidence of regulatory functions only for a small fraction of noncoding DNA (Palazzo & Gregory 2014). Still, genome size affects the cell nucleus size, which affects cell size, and therefore the cell division and development time/rate in plants and animals like invertebrates, amphibians, and fishes, but not amniotes (Bennett 1987; Gregory 2005). As a consequence, genome size might have a negative effect on the phenotypic rate of change in some groups but not in others due to its effect on generation time.
Detailed information of the variable, sources, ploidy level, analytical methods, etc. in the supplementary table "PROCEEDv6.1_species_genome.csv" and its metadata section (Class IV. Data structural descriptors 3).

17. sex: Sex of the individuals measured (male, female, asexual, mixed=male+female, NA(empty cell)=sex not reported in the study).

18. sample1: Identification of Sample 1 (e.g. year, population, identifier given by author(s)). In the case of allochronic studies, Sample 1 should always be the earlier sample. In the case of synchronic studies, sample 1 should always be the source population (e.g. in the case of introductions or range expansions, Sample 1 should be the site “before introduction or expansion”) or the population(s) in the original/ancestral environment condition (e.g. in the case of pollution, sample 1 should be the population in the non-polluted area; in the case of response to introduction, sample 1 should be the population in the area where the introduced species is not present). When the traits are measured in a common garden experiment with different conditions, in more than one common garden location, or in reciprocal transplant experiments, these conditions are noted here (e.g. sample1:"Control origin, control treatment", sample2:"Polluted origin, Control treatment"; sample1: "Control origin, Drought treatment", sample2: "Polluted origin, Drought treatment"; sample1:"Native populations, Jokers Hill common garden", sample2:"Introduced populations, Jokers Hill common garden"; sample1:"Native populations, Montpellier common garden", sample2:"Introduced populations, Montpellier common garden").

19. sample2: Identification of Sample 2 (e.g. year, population, identifier given by author(s)). In the case of allochronic studies, Sample 2 should always be the later sample. In the case of synchronic studies, Sample 2 should always be the later

populations (e.g. in the case of introductions or range expansions, Sample 2 should be the site "after introduction or range expansion") or the population in the new environmental condition (e.g. in the case of pollution, sample 2 should be the population in the polluted area; in the case of response to introduction, sample 2 should be the population in the area where the introduced species is present). When the traits are measured in a common garden experiment with different conditions, or in more than one common garden location, or in reciprocal transplant experiments, these conditions should be log here (e.g. sample1:"Control origin, control treatment", sample2:"Polluted origin, Control treatment"; sample1: "Control origin, Drought treatment", sample2: "Polluted origin, Drought treatment"; sample1:"Native populations, Jokers Hill common garden", sample2:"Introduced populations, Jokers Hill common garden"; sample1:"Native populations, Montpellier common garden", sample2:"Introduced populations, Montpellier common garden").

20. samp1_anc: (yes/no) If sample 1 was from the ancestral population or the population from the ancestral place/source/condition, "samp1_anc"="yes". If there is no ancestral-to-derived relationship between samples, then "samp1_anc"="no".

21. sample1_latitude: Latitude in decimals (to a minimum of three decimal places if possible) of the location of the "sample1" population. Note that South is

negative. If the lat/longs are not given please look up via other studies of the same system, Wikipedia, Google Maps, or OpenStreetMap. You can use these websites to convert from degrees or UTM:

- For URL: http://andrew.hedges.name/experiments/convert_lat_long/
- For UTM: http://www.engineeringtoolbox.com/utm-latitude-longitude-d_1370.html

22. sample1_longitude: Longitude in decimals (to a minimum of three decimal places if possible) of the location of the "sample1" population. Note that West is negative. If the lat/longs are not given please look up via other studies of the same system, Wikipedia, Google Maps, or OpenStreetMap. You can use the above websites to convert from degrees or UTM.

23. sample2_latitude: Same as "sample1_latitude" but for the location of "sample2" populations.

24. sample2_longitude: Same as "sample1_longitude" but for the location of "sample2" populations.

25. coordinate_means: If the coordinates for sample1 and sample2 are the average of coordinates of more than one population (Y) or not (N).

26. coordinate_notes: Source of coordinate information and any other relevant information about the coordinates.

27. design:

- Allochronic: same population/different time points, longitudinal design.
- Synchronic: different populations with known divergence time.

28. genphen: It distinguishes studies measuring individuals that developed in nature (Phenotypic) from those based on measurements of individuals bred in common-garden conditions or applying other quantitative-genetic methods (Genetic). Phenotypic does not mean it is not genetic; we just do not have the information and it does not automatically imply plasticity. Quantitative genetic methods (heritability does not count) are usually based on animal model methods and calculate breeding values which is the genetic contribution to a trait (Wilson et al. 2010). Common garden experiments performed from vegetative (clonal) propagules collected in the field (e.g. tiller, an anatomic unit in grasses) are still "Phenotypic".

29. years: If the study is "design"="Allochronic", the number of years from the start time point to the end time point. If the study is "design"="Synchronic", the

number of years from the divergence point to the date of sampling. If it is less than one year, a decimal is used (number days of study/365).

30. gtime: Generation time expressed in years. We define the generation time as the time (in years) in which a population replaces itself. The supplementary table "PROCEEDv6.1_species_GT.csv" summarizes all the information about generation time for each species, compiled in the current version of PROCEED. See section IV.J.4 of the present document for the full description of the variable, the definitions, criteria, and procedures applied in different situations.

31. gtime_notes: Source of the "gtime" and any other comment related to "gtime" and "generations".

32. generations: The number of generations that have elapsed given the number of years that have elapsed for a given system. It can be calculated as: years/gtime.

33. trait_description: Trait being quantified in the words of the authors of the paper. If the name of the trait is not self descriptive, please include the definition from the paper (e.g. shell slenderness (log10[width] / log10[length])). If units are given, please include. Each trait has its own row.

34. trait_type: Types of traits based on Kingsolver and Diamond (2011) but with some additional categories. Current categories are:

- phenology: the timing of cyclic and seasonal life-history events (e.g. flowering date, laying date, and hatching date). Do not confound with "otherLH". For example, the time spent in a given stage of the life cycle (e.g. larval stage) or the time to reach a given stage of the life cycle (e.g. time to maturity, time to first flower) are life-history traits, not phenology. "data_type" for "phenology" traits is typically a date while for "otherLH" it is time.
- behaviour: "Broadly speaking, animal behavior includes all the ways animals interact with other members of their species, with organisms of other species, and with their environment. Behavior can also be defined more narrowly as a change in the activity of an organism in response to a stimulus, an external or internal cue or combination of cues." (https://www.khanacademy.org/science/ap-biology/ecology-ap/responses-to-the-environment/a/intro-to-animal-behavior)
- physio: traits related to physiology or a biochemical parameter (e.g. enzymatic activity). Example physiology traits include metabolism, bite force, leaf toughness, decomposition rate, stoichiometry (e.g. C:N), concentration or amount (yield) of a given chemical compound in the body (or part of the body), diet (trophic level, diet, food type), severity or

level of affectation by a disease/pathogen/parasite/herbivore (e.g. Fungal infection % of plants, % leaf consumed), dose/concentration/time exposed to a given substance/condition (e.g. pesticide, drought, light, temperature, etc.) needed to produce some effect (e.g. death, flowering, etc.), performance of a standard parasite/herbivore/competitor feeding/competing on/with the focal population (e.g. Pupal weight of the herbivorus *Trichoplusia ni* feeding on native vs introduced populations of *Eschscholzia californica*; or Leaf feeder (*Galerucella pusilla*, L1-Imago) survival, allelopathic effects of *Centaurea maculosa*, *Achillea* biomass ratio with-competition to without-competition).

- growth: a measurement of the change in size (or a proxy of size) in a time period (where the starting point is not the birth, germination or equivalent), or a growth rate (irrespective of the starting point). It can be a relative or an absolute measurement of growth or growth rate. Irrespective of the environmental condition (pollution, stress, competition, herbivory/parasites, fertigated/non-fertigated, etc.), the measurements of growth or growth rate are classified as "growth". If the trait is a change in growth (or another performance measurement) see "response".
- size: Total body mass, or an overall aspect/measurement of body size as a whole (e.g. total length, plant height, thorax with, plant diameter, basal area, number of tillers (grasses)). Size of parts of the body (e.g. tarsus

length, intracranial length, length of the longest leaf, etc.) are not included here but in "othermorphology". In plants, measurements of the whole aboveground size (e.g. aboveground biomass, plant height, plant diameter or basal area, etc.), as well as measurements of the whole belowground size (i.e. root mass) are included as "size". Other morphological features of roots (e.g. length, diameter, etc.) are classified as "othermorphology". The "stem diameter" of tree species is a measurement of "size" but in herbaceous plants, it is " othermorphology ". "size" is not necessarily the final or average body size in a population, the measurements of size at a given age/stage (but not at birth/hatch or equivalent) are also classified as "size" (e.g. Body mass lambs (kg)). Note that, similar situations can be classified differently according to the operational decision of the authors of the primary source of information, e.g.: in a common garden experiment for a short period of time if the authors report final biomass, the trait is classified as "size", but if the authors report growth rate (biomass over time) it is classified as "growth". If the trait is a change in size (or another performance measurement) see "response".

- othermorphology: all remaining morphological traits. This includes the size of parts of the body (e.g. tarsus length, intercranial-length, etc.) or plant (e.g. leaf size). The size of a part of the body at a given age/stage is also "othermorphology". It is independent of the environmental condition

of the measurement, i.e. a measurement of tarsus length (or leaf size, etc.) is always "othermorphology" independently of if it was measured in a controlled environment or in a polluted environment (or in another condition), and so on.

- otherLH: all remaining life history traits that are not classified as phenology or growth (e.g., offspring size, # of offspring, fecundity, survival, parental investment, reproductive effort/allocation, time to reach maturity or any stage in the life cycle). It is independent on the environmental condition of the measurement, i.e. a measurement of survival (or fecundity, etc.) is always "otherLH" independently of is was measured in a controlled environment or in a polluted environment (or in another condition), and so on. If the trait is a change in survival (or another performance measurement) see "response".

- response: response to a number of abiotic and biotic factors. It is the change, relative change, or ratio of a measurement of performance (size, growth, survival, fecundity, etc.) between two conditions (e.g. Size reduction—difference in size between pH 7 and 4, relative to size in pH 7—; Leaf Cpi—[no.leaves_woc - no.leaves_wc]/no.leaves_woc—; Mycorrhizal response [inoculated_biomass-Uninoculated_biomass]/Uninoculated_biomass).

35. data_type: The current categories are:

- linear: a measurement of length with a dimensionality of 1. A ratio between lengths (or a length over another variable) is not a "linear" variable because its dimensionality is not 1. A ratio between linear dimensions (e.g. length over width, or height over diameter), or a linear dimension over another variable (e.g. specific root length (SRL) [mm/mg]) is not "linear" because its dimensionality is not 1 (e.g. SRL=[1D]/[3D], dimensionality -2).
- area (2D): a measurement of area with a dimensionality of 2. A ratio between areas, or an area over another variable (e.g. specific leaf area (SLA) is not "area (2D)" because its dimensionality is not 2 (e.g. SLA=[2D]/[3D], dimensionality -1).
- cube (3D): a measurement of volume or mass or amount of matter with a dimensionality of 3. A ratio between volumes/mass, or a volume/mass over another variable (e.g. "Stem mass ratio (stem mass / whole plant mass)" or "Rhizome mass over length") is not "cube (3D)" because its dimensionality is not 3 (e.g. stem mass / whole plant mass=[3D]/[3D] is a dimensionless proportion; Rhizome mass over length=[3D]/[D], dimensionality is 2 but it is not an "area (2D)" because it depends on the shape of the organ and the density of the tissue, so it is "other").

- count: an amount of discrete units (e.g. number of leaves, clutch size, fecundity when number of offspring or propagules, etc.).
- proportion: the ratio between a part over the total (including that part). It can be expressed as a proportion (0-1), as a percentage (0-100). Some concentrations are also proportions, concentrations expressed as % M/M, % V/V, ppm, mg/g, ug/g (or similar). Molar concentration or µmol/g are not exactly proportions because the numerator and denominator are not in the same units but are still a measurement of the ratio between a part over the total and so, they were classified as ‘proportion’. A ratio between a given amount in a stressing/disturbing condition (e.g. biomass in a toxic environment or in presence of parasite/herbivore or competitors) over a control condition expected to express the maximum potential (e.g. biomass in a non-toxic environment or in absence of parasite/herbivore or competitors), was also considered a "proportion". Similarly, the relative difference between these conditions was also classified as "proportion", because it is a proportional change (e.g. tolerance to herbivory: (damaged plant biomass - undamaged plant biomass) / undamaged plant biomass). The difference is that in the first situation "transf_data"="raw" and in the last one "transf_data"="resid". When the ratio is not a part over the total, this is not a "proportion" (e.g. root to shoot biomass, reproductive over somatic/vegetative biomass), this is a dimensionless ratio (i.e. "ad_ratio").

- time: the time needed for some process or event.
- date: a temporal landmark for a given event in a reference system with an arbitrary starting point (e.g. number of days from January 1st). A time in the day (e.g. hours from midnight) is the same type of trait.
- temperature: temperature in the scale and units reported in the primary source of information (e.g. ºC, ºK, ºF).
- rate: a measurement of speed, an amount over time (e.g., growth rate, metabolic rate (cal/s), etc.). "rate" is a diverse category because the numerator can be "count" (e.g. eggs/day), "linear" (e.g. Growth (mm/day)), "proportion" (Grow(%/d)), "cube (3D)" (e.g. growth rate (pupal mass divided by preadult developmental period) OR Resting Metabolic Rate (mg O_2 h^{-1})), "ad_ratio" (e.g. RGR=ln[m2/m1]/time), "other" ratios (e.g. Stomatal Conductance (mmol m^{-2} s^{-1})), etc. However, even when a trait is called "rate" in the primary source of information is not necessarily a rate according to the current classification (e.g. "pregnancy rate", "survival rate", "germination rate" are "proportion"; "Unit leaf rate (ln [mass_t1 / mass_t0])" is an "ad_ratio"; etc.).
- ad_ratio: the ratio between two independent amounts with the same dimensionality is a dimensionless ratio. It is not a proportion because the numerator is not a component or fraction of the denominator (e.g. shoot:root ratio, C:N ratio, reproductive over vegetative biomass).

- index: ordinal variables and their derivations. However, when a trait is called "index" in the primary source of information it is not necessarily an index according to the current classification. For example, "index of ear size"($[EL \times EW]^{0,5}$) is a called index but it is quantitative variable proportional to the ear size, its dimensionality is 1. We classified this trait as "other". Another example is "Gonadosomatic index (ova mass over body mass)" this is a "proportion" even when called index.

- other: other ratios and other quantitative variables not meeting the previous descriptions.

"data_type" is particularly relevant when computing and comparing darwins (*D*) among different data types. The rate of change darwins is the relative (to mean) rate of change of a given variable (*X*) at a given time (d*t*) (Haldane 1949). Darwins is:

(1) $$D=\frac{d\left(\frac{dX}{X}\right)}{d(t)}$$

by integrating 1/X on dX we obtain:

(2) $$D=\frac{d(\ln(X))}{d(t)}$$

In a discrete interval of time (t), we get:

(3) $$D=\frac{\ln(x_2)-\ln(x_1)}{t}$$

The dimensionality of the trait affects darwins. As an example, if we have three traits total length (*l*), body mass (*m*), and mass of gonads over total body mass (*p*). The relation between these three traits will depend on the allometric relationships, but it would be approximately as follows:

$$m = a \cdot l^3$$

$$p = \frac{b \cdot l^3}{a \cdot l^3} = \frac{b}{a}$$

So, the rate of change in darwins for these traits would be:

$$D(l) = \frac{\ln(l_2) - \ln(l_1)}{t}$$

$$D(m) = \frac{\ln(m_2) - \ln(m_1)}{t} = \frac{\ln(a \cdot l_2^3) - \ln(a \cdot l_1^3)}{t}$$

$$D(m) = \frac{\ln(a) + \ln(l_2^3) - \ln(a) - \ln(l_1^3)}{t} = \frac{3 \cdot \ln(l_2) - 3 \cdot \ln(l_1)}{t} = 3 \cdot \frac{\ln(l_2) - \ln(l_1)}{t}$$

$$D(m) = 3 \cdot D(l)$$

If the allometric relationships remain constant:

$$D(p) = \frac{\ln(p_2) - \ln(p_1)}{t} = \frac{\ln(\frac{b}{a}) - \ln(\frac{b}{a})}{t}$$

$$D(p) = 0$$

As was shown in the previous example, the type of variable, by itself, strongly affects the estimation of evolutionary change in darwins.

36. transf_data: If the trait measurement has been transformed and the means and standard deviations were computed on basis of the transformed data (e.g. body mass expressed as log(g) instead of grams, so the mean is the average of the logarithm of the body mass). The current categories are:

- raw: mean and sd computed from raw data (no transformation).
- ord: the trait is the score in the axis of an ordination analysis (i.e. PCA, CVA).
- arcsin: arc sin ($\sin^{-1}$) transformation.
- arcsin.sqr: angular transformation, i.e. $2\cdot\sin^{-1}$(sqroot(x)).
- resid: residuals of regression or other statistical model (y-ŷ), centered variables (y-ȳ), obsolute (Yt-Yc) or relative difference ([Yt-Yc]/Yc = Yt/Yc -1) between a treatment and a control.
- ln: mean and sd computed from natural log-transformed data. We also include data like ln(x)/ln(y) and ln[x/y]/t
- log10: mean and sd computed from $\log_{10}$-transformed data. We also include data like $\log_{10}$(x)/$\log_{10}$(y) and $\log_{10}$[x/y].

37. data_scale:

- ratio: constant interval with a precise zero.
- interval: constant interval with an arbitrary zero (e.g. temperature, time of day, rescaled data: Principal components or discriminate functions).
- NA (empty cells): undefined scales and ordinal scales (i.e. "data_type"="index") because ordinal scales do not have a constant interval.

Darwins cannot use interval data, but Haldanes can (because it's rescaled units, essentially removing an absolute zero point). Ratio data are actually interval data, but with a clearly defined zero.

38. n1: Sample size for sample 1.

39. n2: Sample size for sample 2.

40. mean1: Mean of the phenotypic trait for a given system for sample 1.

41. mean2: Mean of the phenotypic trait for a given system for sample 2.

42. sd1: The standard deviation around the mean of the phenotypic trait for sample 1. Can be calculated manually if necessary.

43. sd2: The standard deviation around the mean of the phenotypic trait for sample 2. Can be calculated manually if necessary.

44. out_in: Population(s) outside or inside its native range. Of your two data points (samples) to calculate the rates – if one of the datapoints is outside the native range, it is considered “out”. Natural range expansions are considered “in”. You can have an introduction within the native range which would be “in”. Invasive species, with range expansions, would be “out”.

45. urban_disturbance: Category used in Alberti et al. (2017) that classified urban disturbance category (decision tree in Figure S2):

- Biotic: "biotic interactions stem from introductions, and are subcategorized depending on the study organism's ecological role: introduced species, or species in its native range responding to an introduction. Introduced species were further subdivided into instances where the introduced species adapts immediately following introduction, vs adaptation after range expansion (post introduction)".
- Habitat Mod: "changes due to climate change in general, modification of the landscape, or pollution".
- Heterogeneity: "heterogeneity may refer to micro-habitat or micro-climate, and can refer to heterogeneity in space or time".

- Novel: "Novel disturbances were defined as those disturbances to which organisms respond to with novel adaptations, such as the rapid evolution of zinc tolerance".
- Social: "Social interactions refers to disturbances with are direct and intentional result of human agency".

46. disturbance: It is a classification of the environmental change (or driver/disturbance) driving the phenotypic change, based on the categories used by Hendry et al. (2008) and Palkovacs et al. (2012). The current categories are:

- Climate change: this disturbance can often be difficult to clearly identify. A disturbance is classified as "Climate change" when the primary source of information explicitly considers climate change as a potential effect, or when the driver of the system is a climatic event (e.g. *Brassica rapa* (field mustard) changed flowering time after drought in Southern California, USA). Experimental systems manipulating CO2 levels were considered "Pollution" (See "Pollution" definition).
- Hunt_harv: any case of hunting or harvesting of a species by humans.
- Introduction: “when humans transferred a species to a new geographical location, and comparisons were then made between introduced and ancestral populations”. Also, range expansion after introduction (i.e. “when humans introduced a species to a new geographical location, and

the species then spread on its own accord to occupy multiple sites. Comparisons were here made among the self-colonized populations”).

- Pollution: an environmental change is classified as "Pollution" when chemical substances (e.g. heavy metals, pesticides, etc.) are added or released into the environment contaminating, dirtying, and/or making harmful an area (usually air, water, or soil) where the focal species occur. The environmental change resulting from changing the natural concentration of substances present in the environment (e.g. a change in the level of CO_2 in the air or a change in the level of nutrients in the soil) or a physical parameter (e.g. temperature, pH, etc.) is also categorized as "Pollution" even if not harmful to the species being studied.

- Landscape change: It is a habitat modification (different from "Pollution" and the introduction of a new species) in the patches occupied by the population(s) being studied. It can be the creation/restoration of new patches/habitats for the population(s) (e.g. a newly formed freshwater pond for an aquatic species, newly restored habitat), changes in the connectivity among patches/habitats (e.g. the construction of a dam in a river or the construction of a fish ladder, level of fragmentation of the habitat), or a change in the land-use/quality of the patches (e.g. forest clearing, livestock/herbivores inclusion/exclusion, newly irrigation system).

- Response to introductions: when a species (native or alien) is responding to the introduction of a new species. The newly introduced species can be a competitor, a predator, a parasite, a new host or prey, etc. The comparison is established among populations (or times, according to the design) of the responding species (not the newly introduced species). When the responding species is native, "out_in"="In"; when it is alien, "out_in"="Out".
- Other: It is "in situ natural variation" and "Self induced range or host expansion", i.e. when native populations were not subject to an obvious human impact, most studies in this category involve the long-term monitoring of natural populations, such as Darwin’s finches of the Galápagos (Grant & Grant 2002, 2006); also, when new populations were established without any direct human influence, and comparisons were then made among the new populations or among the new and ancestral populations (Examples include birds colonizing new habitats within their native range—Yeh & Price 2004—or new islands outside their native range—Clegg et al. 2002—).

47. env_change:

- ongoing : ongoing environmental change. The environmental change has been occurring through time and the phenotypic change was measured in a temporal window within this process (but not from the starting point).
- novel: novel environmental change. The environmental change has a defined starting point and phenotypic change was measured from this point.

This category applies only to allochronic studies, synchronic are always "env_change"="novel" (because the proposed ancestral state is placed previous to the drivers happening). When "disturbance"="Other", if the driver it is unknown, "env_change"="NA" (empty cell). But in some cases the driver of change is known, like "self induced range expansion", also experimental systems can represent "in situ natural variation" but from a known stating point. In these cases, the value of "env_change" can be chosen.

48. Introductions: This is an arbitrary way of ranking systems with more than one disturbance/driver. It ranks from 0 to 5, with 0 being the disturbance not pertaining to the system at ALL, and 5 being very important to the system. The current method for ranking is:

1 - Range expansions without invasions

2 - Range expansions with invasions (but invasions not specified)

3 - paper specifically investigates response to an invader or introduced species

5 - paper specifically investigates an invader or introduced species

49. Other: This is an arbitrary way of ranking systems with more than one disturbance/driver. It ranks from 0 to 5, with 0 being the disturbance not pertaining to the system at ALL, and 5 being very important to the system. The current method for ranking is:

1 - Paper specifically investigates climate change

5 - Long term monitoring, even for commercially exploited species (e.g. salmonids), but not specifying hunting/harvesting

5 - Paper was in situ natural variation

50. resp_introduced: This is an arbitrary way of ranking systems with more than one disturbance/driver. It ranks from 0 to 5, with 0 being the disturbance not pertaining to the system at ALL, and 5 being very important to the system. The current method for ranking is:

1 - authors note that invasions happened and could be a source of change, but do not explicitly consider it (do not add number to invasions, but do add to landscape change)

4.1 - response to removal of invaders

5 - response to introduced organisms

51. Hunting_Harvesting: This is an arbitrary way of ranking systems with more than one disturbance/driver. It ranks from 0 to 5, with 0 being the disturbance not pertaining to the system at ALL, and 5 being very important to the system. The current method for ranking is:

1 - Looking at herbivory in plants

1 - predation on organisms was specifically considered, but natural predation

2 - predation on organisms was specifically considered, but introduced predation

2.1 predation on organisms specifically considered but REMOVAL of predators due to natural causes

3.1 predation on organisms specifically considered but REMOVAL of predators due to human influences

4 - Species is a commercially or recreationally exploited species (e.g. salmonids), but the study is not specifically considering hunting/harvesting

5 - Paper looks directly at targeted hunting or harvesting by humans

52. landscape_change: This is an arbitrary way of ranking systems with more than one disturbance/driver. It ranks from 0 to 5, with 0 being the disturbance not pertaining to the system at ALL, and 5 being very important to the system. The current method for ranking is:

1 - Paper investigates range expansions w/o invasions

2 - Paper investigates invasions, but not range expansion

2.1 - paper investigates removal of organisms, but not range expansion

3 - Paper investigates range expansions w/ invasions (this includes range expansion into more lakes (for fish) via introduction)

4 - Paper investigates pollution/biocontrol/urbanization

5 - Paper specifically investigates landscape change

53. Pollution: This is an arbitrary way of ranking systems with more than one disturbance/driver. It ranks from 0 to 5, with 0 being the disturbance not pertaining to the system at ALL, and 5 being very important to the system. The current method for ranking is:

1 - pollution is considered as potential cause of observed phenotypic changes

2 - historical fluxes in a pollutant are mentioned in article (e. g. copper flux due to historical copper mining, eutrophication)

5 - Paper specifically investigates pollution/biocontrol

54. climate_change: This is an arbitrary way of ranking systems with more than one disturbance/driver. It ranks from 0 to 5, with 0 being the disturbance not pertaining to the system at ALL, and 5 being very important to the system. The current method for ranking is:

1 - Paper was in situ natural variation

2 - Paper does not mention climate change but talks about environmental variation that can be attributed to climate change (e.g. fluctuation in annual precipitation)

2.1 - time series for commercially exploited species (e.g. salmonids)

3 - Paper specifically investigates range expansion (but not invasions specifically, but if invaders expanded, goes here)

4 - Paper looks at thermal tolerance (natural or induced, e.g. warm water effluent from power plants)

5 - Paper specifically investigates climate change

55. time_series: Point out if this case is related to a time series that can be included in the TimeSeriesDB of PROCEED.

56. tserieshandling: It states how the time series was handled to take two samples to add to the RatesDB.

57. comments: Specify the source of information for each rate (e.g. data source within the paper: figure 3a). Other notes that might be useful to someone looking at the database.

58. DOI: Digital identifier number.

59: reference: This is the reference of the primary source of information, which is given in the following format (all one line): Last name of the first author and year, Journal (standard abbreviations) Volume: Page numbers or article identifier (e.g. Cox 1997, Can. J. Fish. Aquat. Sci. 54: 1159-1165).

60. reference_comments: Comments related to the primary source of information.

61. copyright: If the authors of the manuscript do not want us to share their data or any other information with regards to copyright.

62. who: Who has entered the data (your name).

63. pending_issues: Any unresolved issues that need to be addressed.

64. v5_taxa_fine: This is an old variable kept from previous versions of the database. For future versions, there is no need to code new data entries according to this variable. Currently, this variable has been replaced by "class" and "order" for more precision.

65. v5_Roff_Mousseau: This is an old variable kept from previous versions of the database. For future versions, there is no need to code new data entries according to this variable. The current version of this variable is "trait_type". The last definition of "v5_Roff_Mousseau" was:

Class of traits as determined by Roff and Mosseau (1989).

- Morphological: e.g. body size, wing size, other metric characters.
- Behavioural: e.g. alarm reaction, activity level, sensitive to conditioning.
- Physiological: e.g. oxygen consumption, resistance to heat stress, body temperature.
- Life-history: Fecundity, phenology, viability, survival, development rate, “directly related to fitness”

66. v5_Kingsolver_Diamond: This is an old variable kept from previous versions of the database. For future versions, there is no need to code new data entries

according to this variable. The current version of this variable is "trait_type". The last definition of "v5_Kingsolver_Diamond" was:

"Class of traits as determined by Kingsolver and Diamond (2011). Current classifications are:

- size: Overall aspect of body size (body size, length, mass, PCs for body mass). Size proxies are NOT included here (e.g. tarsus length, intercranial-length, etc.)

- othermorphology: all remaining morphological traits. This includes morphological components that are used as proxies for size (e.g. tarsus length, intercranial-length, etc.)

- phenology: the timing of life-history events (e.g., photoperiod, initial date, peak date, or duration of events such as germination, flowering, laying date, and hatching date)

- otherLH: all remaining life history traits (growth rate, offspring size, # of offspring, survival), if biotic interactions (e.g. competition), then classified as otherLH, if abiotic stressor, classify as physio. For herbivory, see physio – will depend on if it is resistance or tolerance to herbivory.

- behaviour

- physio: metabolism, bite force, growth in stress (abiotic factor), resistance to herbivory (e.g. a chemical defense; tolerance will be based on

trait), diet: trophic level, diet, food type, if both abiotic and biotic interactions, classify as physio.”

67. v5_data_type: This is an old variable kept from previous versions of the database. For future versions, there is no need to code new data entries according to this variable. The current version of this variable is "data_type".

68. v5_driver: This is an old variable kept from previous versions of the database. For future versions, there is no need to code new data entries according to this variable. The current version of this variable is "disturbance".

69. v5_disturbance: This is an old variable kept from previous versions of the database. For future versions, there is no need to code new data entries according to this variable. The current version of this variable is "disturbance".

Class IV. Data structural descriptors 2

C Data set file

C.1 Identity: “PROCEEDv6.1_TimeSeriesDB.csv”

C.2 Size: 12571 rows, 35 columns. 4.7 MB.

C.3 Format and storage mode: CSV file; Language = English; Decimal point = "."; Not available data (NA) = “” (i.e. empty cell).

C.4 Header information: “PROCEEDv6.1_TimeSeriesDB .csv” includes a header with variable names for each column. In section IV.D we describe each variable.

C.5 Alphanumeric attributes: Mixed.

C.6 Special characters/fields: We avoided the use of special characters and comments were placed in specific columns (variables).

C.7 Authentication procedures: There are no authentication procedures to access the data. It is freely available in the Borealis repository (https://doi.org/10.5683/SP3/NXSL3Q).

D Variable information (description by column)

1. uts_ID: Unique identifier for each entry in the TimeSeriesDB. It is a code of the form uts_XXXX, where each X is a character of the ordered sequence: 0, 1, 2, 3, 4, 5, 6, 7, 8, 9, a, b, c, d, e, f, g, h, i, j, k, l, m, n, o, p, q, r, s, t, u, v, w, x, y, z. "uts_ID" are correlative along the whole database, such as "uts_00jz" < "uts_00k0".

2. es_ID: The same identifier as in “PROCEEDv6.1_RatesDB.csv”. Here, it works as an identifier for each individual time series.

3. ref_ID: Same as in “PROCEEDv6.1_RatesDB.csv”. Values linked to the “RatesDB” by the “es_ID”.

4. sys_ID: Same as in “PROCEEDv6.1_RatesDB.csv”. Values linked to the “RatesDB” by the “es_ID”.

5. released_sys: Same as in “PROCEEDv6.1_RatesDB.csv”. Values linked to the “RatesDB” by the “es_ID”.

6. released_es: Same as in “PROCEEDv6.1_RatesDB.csv”. Values linked to the “RatesDB” by the “es_ID”.

7. reference: Same as in “PROCEEDv6.1_RatesDB.csv”. Values linked to the “RatesDB” by the “es_ID”.

8. taxa: Same as in “PROCEEDv6.1_RatesDB.csv”. Values linked to the “RatesDB” by the “es_ID”.

9. phylum: Same as in “PROCEEDv6.1_RatesDB.csv”. Values linked to the “RatesDB” by the “es_ID”.

10. class: Same as in “PROCEEDv6.1_RatesDB.csv”. Values linked to the “RatesDB” by the “es_ID”.

11. order: Same as in “PROCEEDv6.1_RatesDB.csv”. Values linked to the “RatesDB” by the “es_ID”.

12. sp_pub: Same as in “PROCEEDv6.1_RatesDB.csv”. Values linked to the “RatesDB” by the “es_ID”.

13. sp_ncbi: Same as in “PROCEEDv6.1_RatesDB.csv”. Values linked to the “RatesDB” by the “es_ID”.

14. sex: Same as in “PROCEEDv6.1_RatesDB.csv”. Values linked to the “RatesDB” by the “es_ID”.

15. c_value: Same as in “PROCEEDv6.1_RatesDB.csv”. Values linked to the “RatesDB” by the “es_ID”.

16. system: Same as in “PROCEEDv6.1_RatesDB.csv”. Values linked to the “RatesDB” by the “es_ID”.

17. genphen: Same as in “PROCEEDv6.1_RatesDB.csv”. Values linked to the “RatesDB” by the “es_ID”.

18. out_in: : Same as in “PROCEEDv6.1_RatesDB.csv”. Values linked to the “RatesDB” by the “es_ID”.

19. disturbance: Same as in “PROCEEDv6.1_RatesDB.csv”. Values linked to the “RatesDB” by the “es_ID”.

20. env_change: Same as in “PROCEEDv6.1_RatesDB.csv”. Values linked to the “RatesDB” by the “es_ID”.

21. experimental: Same as in “PROCEEDv6.1_RatesDB.csv”. Values linked to the “RatesDB” by the “es_ID”.

22. exp_type: Same as in “PROCEEDv6.1_RatesDB.csv”. Values linked to the “RatesDB” by the “es_ID”.

23. trait_description: Same as in "PROCEEDv6.1_RatesDB.csv". Values linked to the "RatesDB" by the "es_ID".

24. trait_type: Same as in "PROCEEDv6.1_RatesDB.csv". Values linked to the "RatesDB" by the "es_ID".

25. data_type: Same as in "PROCEEDv6.1_RatesDB.csv". Values linked to the "RatesDB" by the "es_ID".

26. transf_data: Same as in "PROCEEDv6.1_RatesDB.csv". Values linked to the "RatesDB" by the "es_ID".

27. data_scale: Same as in "PROCEEDv6.1_RatesDB.csv". Values linked to the "RatesDB" by the "es_ID".

28. gtime: Same as in "PROCEEDv6.1_RatesDB.csv". Values linked to the "RatesDB" by the "es_ID".

29. date_year: This is the calendar year of each observation.

30. time_years: This is the relative year of each time series. Each time series starts from "time_year"=0. "time_years" can be a fraction of a year if the observations were made in intervals shorter than one year (e.g. 0, 0.5, 1, 1.5, 2 in an experimental system).

31. time_generations: This is the relative time of each time series in generations, according to the "time_years" and the "gtime" (from RatesDB linked by the "es_ID"). Each time series starts from "time_generations"=0.

32. n: sample size.

33. mean: mean of the phenotypic trait for a given sample in a time series.

34. sd: the standard deviation, around the mean of the phenotypic trait, for a given sample in a time series.

35. obs: observations and comments.

The R code to link TimeSeriesDB to related values in RatesDB, compute "time_years", "time_generations" and imput "uts_ID" is available in the "PROCEEDv6.1_BasicCode.txt" file.

Class IV. Data structural descriptors 3

E Data set file

E.1 Identity: “PROCEEDv6.1_species_genome.csv”

E.2 Size: 723 rows, 11 columns. 116 kB.

E.3 Format and storage mode: CSV file; Language = English; Decimal point = "."; Not available data (NA) = “” (i.e. empty cell).

E.4 Header information: “PROCEEDv6.1_species_genome.csv” includes a header with variable names for each column. In section IV.F we describe each variable.

E.5 Alphanumeric attributes: Mixed.

E.6 Special characters/fields: We avoided the use of special characters and comments were placed in specific columns (variables).

E.7 Authentication procedures: There are no authentication procedures to access the data. It is freely available in the Borealis repository (https://doi.org/10.5683/SP3/NXSL3Q).

F Variable information (description by column)

1. group: A grouping factor coarser than "taxa". Current categories are: invert (invertebrates), vert (vertebrates), and plant (plants).

2. taxa: This is a coarse classification, not necessarily a clade (monophyletic group). Current categories are: Annelid, Mollusc, Arthropod, Fish, Amphibian, Mammal, Bird, Reptile, Plant.

3. sp_pub: Species name (or subspecific taxon name) of the population(s) being assessed in each case, as was published in the primary source of information.

4. sp_ncbi: Species or subspecific taxon name of the population(s) being assessed in each case, according to the National Center for Biotechnology Information (https://www.ncbi.nlm.nih.gov/Taxonomy/Browser/wwwtax.cgi?mode=Root) in February 2022. Two species were not found in the NCBI database, *Stipa occidentalis* and *Diaptomus sanguineus*, these names were checked in The Plant List (http://www.theplantlist.org/) and WORMS (https://www.marinespecies.org/index.php) respectively. The supplementary table "PROCEEDv6.1_taxonomy.csv" contains the complete list of "sp_pub" and its "sp_ncbi".

5. GS_source: Source of information (citation) for the C-value of a given species.

6. GS_obs: Any relevant information related to the obtaining of the C-value.

7. Cvalue_1C_pg: C-value, i.e. amount of DNA, in picograms (pg), in a haplontic (gamete) nucleus.

8. ploidy: Ploydy level of the species or population, i.e number of chromosome sets in somatic cells of the diplophase (2n).

9. N_chromosomes_2n: Number of chromosomes in somatic cells of the diplophase (2n).

10. method: Determination method of the C-value.

- Fe: Feulgen microdensitometry
- FC_X: flow cytometry
- FC_PI: flow cytometry with propidium iodide
- FC_DAPI: flow cytometry with 4',6'-diamidinophenylindole
- FC_EB: flow cytometry with ethidium bromide
- FC_MI: flow cytometry with mithramycin
- FC_EBO: flow cytometry with ethidium bromide and olivomycin
- MDAPI: microdensitometry with 4',6'-diamidinophenylindole
- RK: reassociation kinetics
- Ch: Chemical extraction
- CIA: unknown

- FIA: Feulgen Image Analysis Densitometry

- SCF: Static cell fluorometry

- BFA: Bulk fluorometric assay

- BCA: Biochemical analysis

- UVM: Ultraviolet microscopy

- GCD: Gallocyanin chrom alum densitometry

- seq: full sequantion

11. congener: Point out if the genome size information belongs to the needed species (i.e. the species in "sp_ncbi"; so, "congener"="no") or if it belongs to a different species from the same genus (so, "congener"="yes").

PLANTS

The main source is:

Leitch IJ, Johnston E, Pellicer J, Hidalgo O, Bennett MD. 2019. Plant DNA C-values Database (Release 7.1). https://cvalues.science.kew.org/

Search options and criteria:

Search for: All Plant C-values

Show estimates: All estimates

Show fields: Genus, Species, Subspecies, Chromosome number, Ploidy level, Estimation method, Prime estimate

C-value: 1C(pg)

The prime genome size estimate was chosen (i.e. the most consistent value obtained under best-practice methods, as defined by Bennett and Smith (1976). If the prime estimation is not linked to Ploidy and Chromosome number, We choose the estimation with these data.

For the not found species, I repeted the search looking for C-value: 1C(Mbp)

When a species name is not found in this database, all synonyms listed in The Plant List (http://www.theplantlist.org/) were checked in the database.

Search of species not found in C-value database

Schoolar Google

Search string:

"Species name"+"DNA content"

ANIMALS

The main source is:

Gregory TR. 2021. Animal Genome Size Database. http://www.genomesize.com.

Search options and criteria:

When multiple estimations are available for a given species, those with not specified (NS) method, standard species, or cell type were ignored.

All estimations with a fitting method, standard species, and cell type were recorded.

In birds variability in genome size is narrow (Andrews 2009). Therefore, when the genome size for a given species was not available, but it was available for some congeners those values were used to average as a proxy of the actual genome size. This was only done for birds. Belonging to the same genus was checked according to taxonomic nomenclature in the NCBI taxonomic database.

To get a preliminary table to load c_value in RatesDB

```
setwd("")
datos <- read.csv("PROCEEDv6.1_species_genome.csv",
na.string="NA")
names <- paste(datos$sp_pub, datos$sp_ncbi, sep="_")
dup <- duplicated(names)
dup2 <- duplicated(names, fromLast=T)
dupp <- dup+dup2
duppm <- dup-dup2
datos2.1 <- datos[which(dupp==0),
c("group","taxa","sp_pub","sp_ncbi","Cvalue_1C_pg")]
datos2.2 <- datos[which(duppm==-
1),c("group","taxa","sp_pub","sp_ncbi","Cvalue_1C_pg")]
datos2.2$Cvalue_1C_pg <- "REVISAR"
datos2 <- rbind(datos2.1, datos2.2)
write.csv(datos2, "species_Cval.csv")
```

Then, check out in “species_Cval.csv” file the cases where Cvalue_1C_pg=”REVISAR” to make a decition according to the criteria described below (When more than one value is available for one species).

When more than one value is available for one species

If there is no difference in ploidy level or chromosome number among records, c-values were averaged.

Records using Feulgen densitometry (Fe), flow citometry (FC_any type), DAPI microdensitometry (MDAPI), Gallocyanin chrom alum densitometry (GDC), or Fulgen image analysis densitometry (FIA) methods were preferred over other methods. So, when available, records using these methods were chosen for averaging.

In birds, if the genome size was estimated from several congeners, the values of the multiple measurements of c-value (if more than one) for each congener were averaged previously, then the average among congeners was computed.

In general, if the genome size was estimated from several subspecies, the value for each subspecies was averaged previously of averaging among them.

In general, but specially in plants, when a species has more than one genome size due to differences in ploidy level or chromosome number, the system was revisited looking for information about particular ploidy levels of the involved

populations for each case. If no information was found, the available c-values were averaged.

Class IV. Data structural descriptors 4

G Data set file

G.1 Identity: "PROCEEDv6.1_taxonomy.csv"

G.2 Size: 435 rows, 8 columns. 35.6 kB.

G.3 Format and storage mode: CSV file; Language = English; Decimal point = "."; Not available data (NA) = "" (i.e. empty cell).

G.4 Header information: "PROCEEDv6.1_taxonomy.csv" includes a header with variable names for each column. In section IV.H we describe each variable.

G.5 Alphanumeric attributes: Mixed.

G.6 Special characters/fields: We avoided the use of special characters and comments were placed in specific columns (variables).

G.7 Authentication procedures: There are no authentication procedures to access the data. It is freely available in the Borealis repository (https://doi.org/10.5683/SP3/NXSL3Q).

H Variable information (description by column)

1. sp_pub: Species name (or subspecific taxon name) of the population(s) being assessed in each case, as was published in the primary source of information.

2. common: Species common (non-scientific) name (or subspecific taxon name) of the population(s) being assessed in each case, as was published in the primary source of information.

3. taxa: This is a coarse classification, not necessarily a clade (monophyletic group). Current categories are: Annelid, Mollusc, Arthropod, Fish, Amphibian, Mammal, Bird, Reptile, Plant.

4. phylum: Taxonomic clade of rank "phylum" including the studied species, according to the National Center for Biotechnology Information (https://www.ncbi.nlm.nih.gov/Taxonomy/Browser/wwwtax.cgi?mode=Root) last retrieved May 2022.

5. class: Taxonomic clade of rank "class" including the studied species, according to the National Center for Biotechnology Information (https://www.ncbi.nlm.nih.gov/Taxonomy/Browser/wwwtax.cgi?mode=Root) last retrieved May 2022.

6. order: Taxonomic clade of rank "order" including the studied species, according to the National Center for Biotechnology Information

(https://www.ncbi.nlm.nih.gov/Taxonomy/Browser/wwwtax.cgi?mode=Root) last retrieved May 2022.

7. sp_ncbi: Species or subspecific taxon name of the population(s) being assessed in each case, according to the National Center for Biotechnology Information (https://www.ncbi.nlm.nih.gov/Taxonomy/Browser/wwwtax.cgi?mode=Root) in February 2022. Two species were not found in the NCBI database, *Stipa occidentalis* and *Diaptomus sanguineus*, these names were checked in The Plant List (http://www.theplantlist.org/) and WORMS (https://www.marinespecies.org/index.php) respectively.

8. sp_pub.ncbi: It is a classification factor that states if the given name ("sp_pub") and the verified name ("sp_ncbi") are equal ("eq") or not ("not_eq").

Class IV. Data structural descriptors 5

I Data set file

I.1 Identity: “PROCEEDv6.1_species_GT.csv”

I.2 Size: 1690 rows, 5 columns. 269.7 kB.

I.3 Format and storage mode: CSV file; Language = English; Decimal point = "."; Not available data (NA) = “NA”.

I.4 Header information: “PROCEEDv6.1_species_GT.csv” includes a header with variable names for each column. In section IV.J we describe each variable.

I.5 Alphanumeric attributes: Mixed.

I.6 Special characters/fields: We avoided the use of special characters and comments were placed in specific columns (variables).

Authentication procedures: There are no authentication procedures to access the data. It is freely available in the Borealis repository (https://doi.org/10.5683/SP3/NXSL3Q).

J Variable information (description by column)

1. taxa: This is a coarse classification, not necessarily a clade (monophyletic group). Current categories are: Annelid, Mollusc, Arthropod, Fish, Amphibian, Mammal, Bird, Reptile, Plant.

2. sp_ncbi: Species or subspecific taxon name of the population(s) being assessed in each case, according to the National Center for Biotechnology Information (https://www.ncbi.nlm.nih.gov/Taxonomy/Browser/wwwtax.cgi?mode=Root) in February 2022. Two species were not found in the NCBI database, *Stipa occidentalis* and *Diaptomus sanguineus*, these names were checked in The Plant List (http://www.theplantlist.org/) and WORMS (https://www.marinespecies.org/index.php) respectively. The supplementary table "PROCEEDv6.1_taxonomy.csv" contains the complete list of "sp_pub" and its "sp_ncbi".

3. sp_pub: Species name (or subspecific taxon name) of the population(s) being assessed in each case, as was published in the primary source of information.

4. gtime: Generation time expressed in years. There are several definitions of the generation time. For our purposes, we define the generation time as the time (in years) in which a population replaces itself.

- For species with non-overlapping generations or species whose individuals reproduce only once in life (e.g. monocarpic plants), the average age at maturity is appropriate.
- For species with non-overlapping generations or species whose individuals reproduce only once in life, with the average age at maturity

shorter than one year but experiencing a period of non reproduction in the year (e.g. diapause in mosquitoes, plants with dormant seeds), the generation time was computed as the reciprocal of the number of generations per year (1/GPY). If the generation time is less than one year, the number will be a decimal (# days/365 or # months/12).

- For species with overlapping generations, individuals reproducing multiple times in life and age-structured populations, the age at which members of a given cohort are expected to reproduce is an accurate proxy. It can be computed from a life table as:

$$GT=\frac{\sum_{i=1}^{n} x_i \cdot l_{x_i} \cdot m_{x_i}}{\sum_{i=1}^{n} l_{x_i} \cdot m_{x_i}}$$

Where x_i is the age (or the mean for an age class), l_{xi} is the proportion of survivors (from the age=0) to a given age (x_i), and m_{xi} is the average fecundity of the individuals at a given age (x_i). A similar measurement is the average difference in age between parent and offspring, but it requires a stable age structure in the population.

- For species with overlapping generations, individuals reproducing multiple times in life and stage-structured populations, the time it takes for the population to grow by a factor of its net reproductive rate (R_0) is an accurate proxy. It can be computed as:

$$\mathrm{GT} = \frac{\ln(R_0)}{r}$$

such as:

$$e^{r \cdot \mathrm{GT}} = R_0$$

Where r=ln(λ), and λ is the finite population growth rate. These parameters can be computed from the transition matrix.

Ultimately, the generation time is used to compute the number of elapsed generations between Sample 1 and Sample 2. Ideally, we want the specific generation time for the population (because the generation time can vary among populations). When the authors from the primary source of information provide an estimation of the generation time, or number of generations per year or total number of generations, this information was used. If this is unavailable, the following steps can be taken:

- Query the author directly (see contacting authors).
- Web of Science or Google Scholar search looking for the generation time.
- For marine species, consult the "The Marine Life Information Network" (https://www.marlin.ac.uk/). Then, search for your species, in the "Life history" table you might find the "generation time".
- An option for fish is to consult the FishBase (https://www.fishbase.se/search.php).

- The databases COMPADRE (https://compadre-db.org/) and COMADRE (https://compadre-db.org/Data/Comadre) provide population transition matrices for plants and animal species respectively. From these matrices, the generation time can be computed as the time it takes for the population to grow by a factor of its net reproductive rate by using the R package "popbio" (Stubben & Milligan 2007). Generation time from Caswell (2001) eq. 5.73:

$$GT = \frac{\ln(R_0)}{\ln(\lambda)}$$

Example using "popbio" in R:

```
#Fmtr: fecundity matrix
#Umtr: survival matrix
#Amtr: (full) transition matrix
#R0:
Rmtr <- Fmtr%*%fundamental.matrix(Umtr)$N
R0 <- eigen(Rmtr)$value[1]
#λ:
lmd <- pop.projection(Amtr, rep(100, nrow(Amtr)),
100)$lambda
#GT:
log(R0)/log(lmd)
```

- If the generation time, a table of life, a transition matrix, or the demographic information to build a table of life are not available, you can look for detailed information on the life cycle and the time and probability for each stage and transition. Based on this information you can produce an educated guess of the actual generation time.
- Look at the Encyclopedia of Life website (http://eol.org/). Then, (i) search for your species, (ii) click on read full entry, (iii) look for the box on reproduction – there might be information on age at maturity. For animals with a long gestation period (e.g. bears), sum this gestation period to the age at maturity. Then, the generation time is the earliest age when they give birth, not when they become sexually mature. Even this is (most likely) an underestimation of the actual generation time.

5. gtime_notes: Source of the "gtime" and any other comment related to "gtime" and "generations".

Class IV. Data structural descriptors 6

K Data set file

K.1 Identity: “PROCEEDv6.1_BasicCode.txt”

K.2 Size: 17.7 kB.

K.3 Format and storage mode: plain text, Unix line ending format.

K.4 Header information: “PROCEEDv6.1_BasicCode.txt” does not include a header.

K.5 Alphanumeric attributes: Text

K.6 Special characters/fields: We avoided the use of special characters.

Authentication procedures: There are no authentication procedures to access the data. It is freely available in the Borealis repository (https://doi.org/10.5683/SP3/NXSL3Q).

L Variable information.

This file compiles R code for some basic handling of PROCEEDv6.1 and examples to perform some basic analyses and graphics.

Class V. Supplemental descriptors

A Data acquisition

A.1 Data acquisition methods: The data was extracted from primary sources of information, like journal papers or technical reports of governmental agencies.

B Quality assurance/quality control procedures: As data was extracted from other souces of information, no data was assumed to be an outlier *a priori*.

C Related materials: no additional materials is related to PROCEED.

D Computer programs and data-processing algorithms: All methodological desicions are described in the variable descriptions, as well as the scripts used for handling procedures.

E Archiving

E.1 Archival procedures: PROCEED v6.1 is archived, for long-term storage in the Borealis repository (https://doi.org/10.5683/SP3/NXSL3Q). The previous versions, as well as the live version of the datasets, are available on GitHub (https://github.com/photopidge/PROCEED).

F Publications and results, lists of publications resulting from PROCEED:

- Sanderson et al. 2023.
- Sanderson et al. 2021.
- Gorné & Díaz 2019.
- Gorné & Díaz 2017.
- Alberti et al. 2017.
- Gotanda et al. 2015.

- Palkovacs et al. 2012.

- Westley 2011.

- Crispo et al. 2010.

- Darimont et al. 2009.

- Hendry et al. 2008.

- Kinnison & Hendry 2001.

- Hendry & Kinnison 1999.

G History of data set usage

G.1 Data set update history: A full record of the changes and editions made in PROCEED up to version 6 (PROCEEDv6_changes_record.pdf) and version 6.1 (PROCEEDv6.1_changes_record.pdf) are available in the Borealis repository (https://doi.org/10.5683/SP3/NXSL3Q).

Literature Citations

- Abrahamson S, Bender MA, Conger A D & Wolff S. 1973. Uniformity of radiation-induced mutation rates among different species. Nature, 245(5426): 460–462.
- Alberti M, Correa C, Marzluff J, Hendry AP, Palkovacs EP, Gotanda KM, Hunt V, Apgar TM & Zhou Y. 2017. Global urban signatures of phenotypic change in animal and plant populations. Proc. Natl. Acad. Sci., 114(34): 8951–8956.
- Andrews CB. 2009. Constraints on genome size in birds (Doctoral dissertation, University of Guelph).
- Barrett RDH & Schluter D. 2008. Adaptation from standing genetic variation. Trends in Ecology and Evolution, 23(1): 38–44.
- Bennett MD. 1987. Variation in genomic form in plants and its ecological implications. New Phytologist, 106(1), 177–200.
- Bennett MD & Smith JB. 1976. Nuclear DNA amounts in angiosperms. Philosophical Transactions of the Royal Society of London Series B: Biological Sciences, 274(933): 227–274.
- Caswell H. 2001. Matrix Population Models: Construction, Analysis, and Interpretation. Sinauer, Sunderland, MA, second edition.
- Carroll SP, Loye JE, Dingle H, Mathieson M, Famula TR & Zalucki MP. 2005. And the beak shall inherit–evolution in response to invasion. Ecology letters, 8(9): 944-951.
- Caswell H. 2001. Matrix population models: construction, analysis and interpretation (2nd ed.). Sinauer.

- Clegg SM, Degnan SM, Moritz C, Estoup A, Kikkawa J & Owens IP. 2002. Microevolution in island forms: the roles of drift and directional selection in morphological divergence of a passerine bird. Evolution, 56(10): 2090-2099.
- Cox SP & Hinch SG. 1997. Changes in size at maturity of Fraser River sockeye salmon (*Oncorhynchus nerka*) (1952 1993) and associations with temperature. Canadian Journal of Fisheries and Aquatic Sciences, 54(5): 1159-1165.
- Crispo E, DiBattista JD, Correa C, Thibert-Plante X, McKellar AE, Schwartz AK, Berner D, De Leon LF & Hendry AP. 2010. The evolution of phenotypic plasticity in response to anthropogenic disturbance. Evolutionary Ecology Research, 12(1): 47-66.
- Darimont CT, Carlson SM, Kinnison MT, Paquet PC, Reimchen TE, Wilmers CC. 2009 Human predators outpace other agents of trait change in the wild. Proc. Natl. Acad. Sci. U. S. A., 106(3): 952–954.
- Gingerich PD. 1993. Quantification and comparison of evolutionary rates. American Journal of Science, 293(A): 453-478.
- Grant PR & Grant BR. 2002. Unpredictable evolution in a 30-year study of Darwin's finches. Science, 296(5568): 707-711.
- Grant PR & Grant BR. 2006. Evolution of character displacement in Darwin's finches. science, 313(5784): 224-226.
- Gregory TR. 2001. Coincidence, coevolution, or causation? DNA content, cellsize, and the C-value enigma. Biological Reviews, 76(1): 65–101.
- Gregory TR. 2005. The evolution of the genome. London: Elsevier Academic Press.

- Gregory TR. 2021. Animal Genome Size Database. http://www.genomesize.com

- Gorné LD & Díaz S. 2017. A novel meta-analytical approach to improve systematic review of rates and patterns of microevolution. Ecology and Evolution, 7(15): 5821–5832.

- Gorné LD, Díaz S. 2019. Meta-analysis shows that rapid phenotypic change in Angiosperms in response to environmental change is followed by stasis. The American Naturalist, 194(6): 840–853.

- Gotanda KM, Correa C, Turcotte MM, Rolshausen G & Hendry AP. 2015. Linking macro-trends and micro-rates: re-evaluating micro-evolutionary support for Cope's rule. Evolution, 69(5): 1345–1354.

- Haldane JBS. 1949. Suggestions as to quantitative measurement of rates of evolution. Evolution, 3(1): 51-56.

- Hendry AP, Farrugia TJ & Kinnison MT. 2008. Human influences on rates of phenotypic change in wild animal populations. Molecular Ecology, 17(1): 20–29.

- Hendry AP & Kinnison MT. 1999. The pace of modern life: measuring rates of micro-evolution. Evolution, 53(6): 1637-1653.

- Kardos M & Luikart G. 2021. The genetic architecture of fitness drives population viability during rapid environmental change. The American Naturalist, 197(5): 511– 525.

- Kingsolver JG & Diamond SE. 2011. Phenotypic selection in natural populations: what limits directional selection?. The American Naturalist, 177(3): 346-357.

- Kinnison MT & Hendry AP. 2001. The pace of modern life II: from rates of contemporary microevolution to pattern and process. Genetica, 112(1): 145–164.
- Knight CA, Molinari NA & Petrov DA. 2005. The large genome constraint hypothesis: Evolution, ecology and phenotype. Annals of Botany, 95(1): 177–190.
- Lacy RC. 1997. Importance of genetic variation to the viability of mammalian populations. Journal of Mammalogy, 78(2): 320–335.
- Lande R & Shannon S. 1996. The role of genetic variation in adaptation and population persistence in a changing environment. Evolution, 50(1): 434–437.
- Leaver SD & Reimchen TE. 2012. Abrupt changes in defence and trophic morphology of the giant threespine stickleback (Gasterosteus sp.) following colonization of a vacant habitat. Biological Journal of the Linnean Society, 107(3): 494-509.
- Leitch IJ, Johnston E, Pellicer J, Hidalgo O & Bennett MD. 2019. Plant DNA C-values Database (Release 7.1). https://cvalues.science.kew.org/
- Lerman A. 1965. On rates of evolution of unit characters and character complexes. Evolution, 19(1): 16-25.
- Lynch M. 1990. The rate of morphological evolution in mammals from the standpoint of the neutral expectation. The American Naturalist, 136(6): 727–741.
- Macnair MR. 1991. Why the evolution of resistance to anthropogenic toxins normally involves major gene changes: The limits to natural selection. Genetica, 84(3): 213– 219.
- Martin SL & Husband BC. 2012. Whole Genome Duplication Affects Evolvability of Flowering Time in an Autotetraploid Plant. PLoS ONE, 7(9): e44784.

- Meyerson LA, Pyšek P, Lučanová M, Wigginton S, Tran CT & Cronin JT. 2020. Plant genome size influences stress tolerance of invasive and native plants via plasticity. Ecosphere, 11(5): e03145.- Mosseau TA & Roff DA. 1989. Adaption to seasonality in a cricket: patterns of phenotypic and genotypic variation in body size and diapause expression along a cline in season length. Evolution, 43(7): 1483-1496.
- Oomen RA, Kuparinen A & Hutchings JA. 2020. Consequences of single-locus and tightly linked genomic architectures for evolutionary responses to environmental change. Journal of Heredity, 111(4): 319– 332.
- Palazzo AF & Gregory TR. 2014. The Case for Junk DNA. PLoS Genetics, 10(5): e1004351.
- Palkovacs EP, Kinnison MT, Correa C, Dalton CM & Hendry AP. 2012. Fates beyond traits: Ecological consequences of human-induced trait change. Evolutionary Applications 5(2): 183–191.
- Sanderson S, Beausoleil MO, O'Dea RE, Wood ZT, Correa C, Frankel V, Gorné LD, Haines GE, Kinnison MT, Oke KB, Pelletier F, Pérez-Jvostov F, Reyes-Corral WD, Ritchot Y, Sorbara F, Gotanda KM, Hendry AP. 2021. The pace of modern life, revisited. Molecular Ecology, 31(4): 1028–1043.
- Sanderson S, Bolnick DI, Kinnison MT, O'Dea RE, Gorné LD, Hendry AP, Gotanda KM. 2023. Contemporary changes in phenotypic variation, and the potential consequences for eco-evolutionary dynamics. Ecology Letters, 26(S1): S127–S139.

- Shanmugam A, Nagarajan A & Pramanayagam S. 2017. Non-coding DNA – a brief review. Journal of Applied Biology & Biotechnology, 5(05): 42–47.
- Sparrow AH, Cuany RL, Miksche JP & Schairer LA. 1961. Some factors affecting the responses of plants to acute and chronic radiation exposures. Radiation Botany, 1(C): 10–34.
- Stamp MA & Hadfield JD. 2020. The relative importance of plasticity versus genetic differentiation in explaining between population differences; a meta-analysis. Ecology Letters, 23(10): 1432–1441.
- Stubben C & Milligan B. 2007. Estimating and analyzing demographic models using the popbio package in R. Journal of Statistical Software 22(11): 1-23.
- Vinogradov AE. 2003. Selfish DNA is maladaptive: Evidence from the plant Red List. Trends in Genetics, 19(11): 609–614.
- Vinogradov AE. 2004. Genome size and extinction risk in vertebrates. Proceedings of the Royal Society B: Biological Sciences, 271(1549): 1701–1705.
- Westley PAH. 2011. What invasive species reveal about the rate and form of contemporary phenotypic change in nature. The American Naturalist, 177(4): 496–509.
- Wilson AJ, Reale D, Clements MN, Morrissey MM, Postma E, Walling CA, Kruuk LEB, & Nussey DH. 2010. An ecologist's guide to the animal model. Journal of Animal Ecology, 79(1): 13-26.
- Wright S. 1968. Evolution and the genetics of populations. I. Genetic and biometric foundations. Univ. of Chicago Press, Chicago, USA.

- Yeh PJ & Price TD. 2004. Adaptive phenotypic plasticity and the successful colonization of a novel environment. The American Naturalist, 164(4): 531-542.
- Zuckerkandl E. 2002. Why so many noncoding nucleotides? The eukaryote genome as an epigenetic machine. Genetica, 115(1): 105–129.
